# A simple cell-cycle control system in *Marchantia polymorpha* provides a framework for understanding plant cell proliferation

**DOI:** 10.1093/plcell/koag103

**Published:** 2026-04-09

**Authors:** Facundo Romani, Ignacy Bonter, Marius Rebmann, Go Takahashi, Fernando Guzman-Chavez, Francesco De Batté, Yuki Hirakawa, Jim Haseloff

**Affiliations:** Department of Plant Sciences, University of Cambridge, Cambridge CB2 3EA, United Kingdom; Department of Plant Sciences, University of Cambridge, Cambridge CB2 3EA, United Kingdom; Department of Plant Sciences, University of Cambridge, Cambridge CB2 3EA, United Kingdom; Graduate School of Integrated Sciences for Life, Hiroshima University, Higashi Hiroshima, Hiroshima 739-8526, Japan; Faculty of Chemistry, Food and Biotechnology Department, Universidad Nacional Autónoma de México UNAM, Ciudad Universitaria, Mexico City 04510, México; Department of Plant Sciences, University of Cambridge, Cambridge CB2 3EA, United Kingdom; Graduate School of Integrated Sciences for Life, Hiroshima University, Higashi Hiroshima, Hiroshima 739-8526, Japan; Department of Plant Sciences, University of Cambridge, Cambridge CB2 3EA, United Kingdom

## Abstract

Eukaryotic cell division is controlled by cyclins and cyclin-dependent kinases (CDKs). The high number of cyclin-CDK pairs in flowering plants hinders functional analysis due to redundancy, and how this system might have worked in early land plant ancestors remains unresolved. Our phylogenetic analysis showed that non-seed plants have a simple system of cell cycle genes, suggesting that the complexity in seed plants is a derived feature. To explore simpler systems, we studied the liverwort *Marchantia polymorpha*, which possesses a reduced, nonredundant set of core cell cycle genes. Single-cell RNA-seq and live imaging of fluorescent reporters revealed phase-specific expression of cell cycle genes during cell division, characterized by 1 predominant cyclin per phase in the vegetative gametophyte, with limited overlap at transitions. Live imaging of tagged cyclins indicated that protein turnover and localization contribute to phase specificity. Functional studies revealed that MpCYCD;1 is sufficient to promote cell cycle re-entry, while overexpression of MpCYCA and MpCYCB;1 causes growth arrest, consistent with their roles in the G1, S, and G2/M transitions. Our findings reveal conserved features of cell cycle control across eukaryotes and the ancestral state of land plants. Marchantia thus provides a powerful framework for understanding multicellular proliferation and its evolution, with the potential for engineering plant growth and development.

## Teaser

The liverwort Marchantia uncovers how cyclin proteins sequentially control cell division phases, revealing ancestral molecular mechanisms that shaped the evolution of growth across land plants.

## Introduction

Eukaryotic organisms have evolved sophisticated mechanisms to control cell proliferation. While cell-cycle control shares common features across eukaryotes, with a conserved set of molecular mechanisms, each lineage of plants, animals, and fungi operates different versions during the establishment of their varied body plans (Harashima et al. 2013). Understanding these regulatory networks is a central question for developmental biology and the evolution of development. This knowledge will be necessary for rational engineering of growth and neo-organogenesis in crop plants and could promote radical advances in agricultural biotechnology (Brophy et al. 2018).

The eukaryotic mitotic cell cycle progresses through G1 (gap 1), S (synthesis), and G2 (gap 2) phases to mitosis (M) (Mironov et al. 1999; Krylov et al. 2003; Harashima et al. 2013). The transition between phases is governed by a relay of cyclins that activate cyclin-dependent kinases (CDKs) to ensure the proper coordination of genome replication and chromosomal segregation. Group I cyclins are unequivocally associated with cell cycle regulation (Martinez-Alonso and Malumbres 2020), following an archetypical sequence: D-type cyclins regulate the G1/S transition, A-type cyclins are associated with the S-phase, and B-type cyclins with the G2/M transition (Inze and De Veylder 2006; Komaki and Sugimoto 2012). The interaction of cyclins with their CDK partners phosphorylate the retinoblastoma (RB) protein and triggers the transduction of signals necessary for the division of cells. The regulation of these networks is not only crucial for controlling the rate of cell division but also for maintaining the appropriate balance between proliferation and differentiation and is essential for functional integrity, proper organ formation, and integration of environmental responses (Inze and De Veylder 2006; Gutierrez 2022).

While the canonical organization of phase-specific cyclins/CDKs is well portrayed in literature and textbooks, most model organisms have additional or missing sets of genes that complicate the understanding of this system. For example, animals have E-type cyclins, another G1-specific cyclin that overlaps with D-type, and yeast do not have a true A-type cyclin ortholog. This question is of special importance in plants where the high complexity of cyclin and CDK regulation, especially in flowering plants, made it challenging to disentangle their precise functional roles in the progression of the cell cycle in plants (Gutierrez 2022). Plants also exhibit unique cell cycle features, such as a plant-specific CDKB expressed during G2/M (Gutierrez 2009).

The Arabidopsis genome displays a large repertoire of cell cycle genes, including 31 Group I cyclins. Among the 10 D-type cyclins, some peak at G1, others at S, and others at G2/M, and only *CYCD3;3* and *CYCD5;1* follow the canonical expression pattern (Menges et al. 2005, 2006). Similarly, expression of the A-type *CYCA3* subfamily peaks at S, while *CYCA1* and *CYCA2* peak at G2/M (Menges et al. 2005). Only B-type cyclins and CDKB genes are consistently expressed at G2/M (Menges et al. 2005). This diversity of expression patterns could be a consequence of gene redundancy and cell cycle specialization. The roles of individual cyclins are not always clear compared with the canonical system described in animals and yeast. This has hindered our ability to understand many fundamental aspects of the cell cycle regulation and its evolution in plants.

To understand the plant cell cycle regulation, it is crucial to employ diverse model species. Research in green algae like *Chlamydomonas reinhardtii* has provided critical insights into the fundamental architecture of the cell cycle (Cross and Umen 2015; Tulin and Cross 2015; Atkins and Cross 2018; Breker et al. 2018; Cross 2020), including conserved regulation of the anaphase-promoting complex by the plant specific CYCB/CDKB complex (Pecani et al. 2022). However, Chlamydomonas exhibits an uncommon multiple-fission cell cycle, that consists of a long G1 phase followed by successive rounds of S phases and M (Cross and Umen 2015), making it hard to directly compare with land plant species. Nonvascular plants offer a promising opportunity to study the cell cycle in early divergent plants, with different cellular body plans. Studies of the moss *Physcomitrium patens* have revealed both conserved and unique aspects of plant cell cycle regulation (Schween et al. 2008; Ishikawa et al. 2011; Peramuna et al. 2023). However, the large repertoire of cyclins and CDKs in Physcomitrium creates challenges for understanding due to functional redundancy.

The model liverwort *Marchantia polymorpha* features a reduced set of cell-cycle regulators (Bowman et al. 2017). The simplicity of these regulatory circuits and experimentally accessible features of the organism (Bowman et al. 2022; Romani et al. 2024) positions Marchantia as a key organism to study to understand the evolution of the cell-cycle control system in a land plant without the confounding effects of gene redundancy and subfunctionalization. However, functional studies associated with the cell cycle in this species are very limited (Nishihama et al. 2015; Hernandez-Garcia et al. 2021).

In this work, we made a comprehensive phylogenetic analysis of cell cycle genes across plants and other eukaryotes and confirmed that Marchantia retains a minimal set of regulators. Expression profiles and functional analyses allowed us to elucidate the organization of the core cell cycle regulation genes that operate in Marchantia and compare this system with its counterparts in other eukaryotes, with 1 active cyclin in each phase. Misexpression of cyclin genes in the Marchantia vegetative gametophyte revealed that Mp*CYCD;1* is a rate-limiting factor for cell proliferation, promoting dedifferentiation, while Mp*CYCB;1* and Mp*CYCA* cause cell cycle arrest. On the other hand, MpCYCB;2 is a putative pseudogene and MpCYCD;1 is not a canonical D-type cyclin, without significant impact on gametophyte growth. Overall, we see that Marchantia has a streamlined and minimalistic cell-cycle control system that will be useful for study of cell proliferation and as a prototype for reprogramming plant growth and development.

## Results

### Conservation and expansion of cell cycle genes in eukaryotes and plants

**Figure 1:**
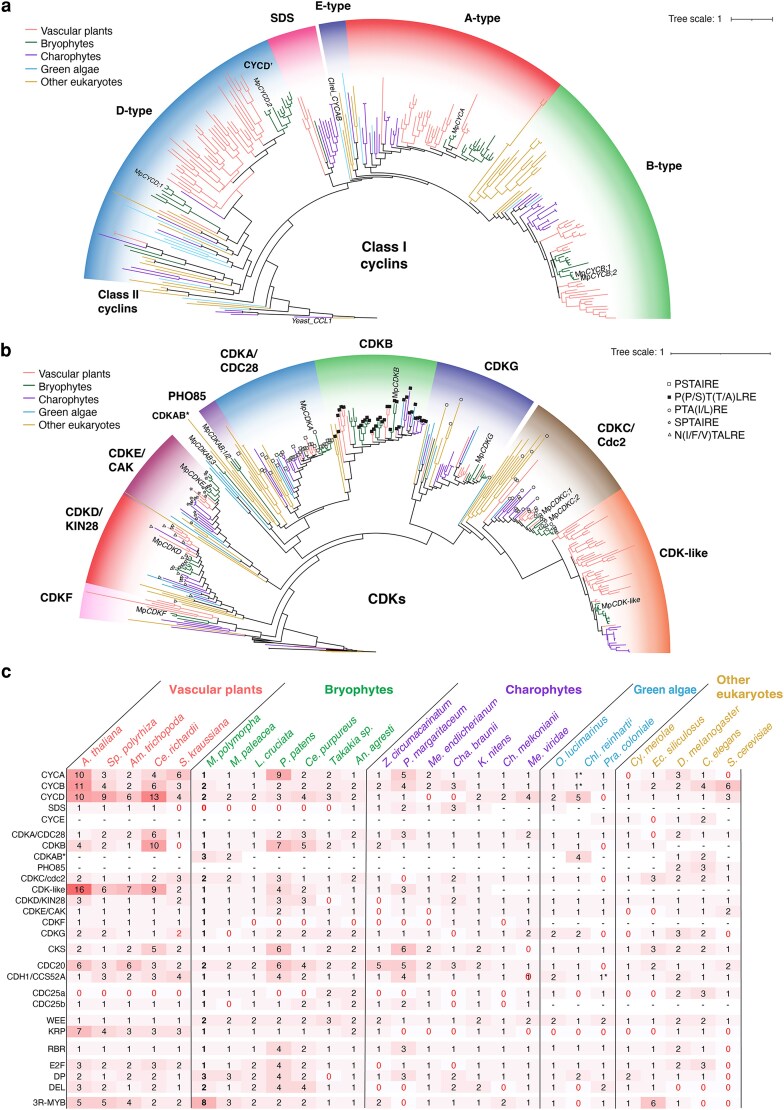
Phylogenetic analysis of core cell cycle genes across eukaryotes. a-b) Maximum likelihood phylogenetic analysis of cyclins across representative plant and eukaryotic species. a) and CDKs b). In each diagram, subfamilies are indicated with different shaded background colors. Branches are colored according to species in each clade as shown in the figure legends. Conserved signature motifs across CDKs are indicated in each node as shown in the figure key on the right. Full phylogenies and details are available at Dataset S1. Scale bars correspond to substitutions per site. c) Summary of the number of cell cycle genes across eukaryotic species. Background color indicates the total number of genes in darker red. Hyphens indicate absence, while red zeros indicate potential losses. *Chlamydomonas reinhardtii has an extra divergent *CYCAB1* (*Cre08.g370401*).

To investigate the core regulation of cell cycle regulation in land plants, we conducted a phylogenetic analysis of cell cycle genes in a broad range of 27 eukaryotic species, including yeast, animals, brown and red algae, charophytes, bryophytes, lycophytes, ferns, and flowering plants (Fig. 1). We focused on gene families that have been described as fundamental components of the cell cycle control machinery, including: Group I cyclins, CDKs, CYCLIN-DEPENDENT KINASES REGULATORY SUBUNIT (CKS), RETINOBLASTOMA-RELATED (RBR) proteins, the CIP/KIP-related protein (KRP) and WEE repressors, CDC25, the co-activators of the Anaphase Promoting Complex/Cyclosome (APC/C), and associated transcription factors (TFs) from the E2F, DP, DEL, and 3R-MYB gene families.

A-, B-, and D-type cyclins are well conserved across eukaryotes, except fungi, which only display 2 types of cyclins, normally referred as *Ccl* and *Cln* classes, analogous to B-type and the D-type (Fig. 1a and c). E-type cyclins are specific to animals, with potential presence in other green algal lineages but absent in land plants (Fig. 1a and b). SOLO DANCERS (SDS) cyclins are a group of plant-specific cyclins; SDS cyclins are associated with meiosis (Azumi et al. 2002) and likely originated in charophytic algae, but those genes were subsequently lost in some nonseed plants (Fig. 1c). The distribution of genes is consistent with previous phylogenetic analysis (Ma et al. 2013). Interestingly, gene duplication of the D-type cyclins appears to have given rise to a new clade of bryophyte-specific cyclins that we named CYCD’ (Fig. 1a and c).

CDKAs and CDKBs are the main partners that interact with cyclins to help govern cell cycle progression in plants. CDKA, homologous to yeast CDC28, is conserved across eukaryotes with the hallmark PSTAIRE motif (Fig. 1b and c, Dataset S1). The G2/M-specific CDKB is ubiquitously present in all green plants (Fig. 1b and c). In nonflowering plants, they are associated with the PSTALRE motif (Fig. 1b) that is well conserved across the family, as opposed to the P(P/S)T(A/T)LRE in flowering plants (Joubes et al. 2000). The only known case where this motif is found in CDKBs outside the green algae is in the brown algae *Ectocarpus siliculosus*, which contains 2 divergent CDKB-like CDKs with the PSTALRE motif (Fig. 1b).

Other CDKs can act as activators within the cell cycle phosphorylation cascade (Joubes et al. 2000). CDKC (homologous to yeast CTK), CDKD (KIN28), CDKE (CAK1/SSN3), and CDKG are conserved across eukaryotes, with some lineage-specific losses (Fig. 1b and c). Notably, the CDKC subfamily has expanded in plants, forming a distinct streptophyte-specific CDK-like clade, whose function is largely unknown (Joubes et al. 2000). Among the remaining clades, CDKF is found in all streptophytes, and the PHO85 clade, which belongs to the CDKA/CDC28 super-family, is conserved in yeast and animals but not found in most other eukaryotes (Liu and Kipreos 2000). Divergent CDKAB members form a paraphyletic group in scattered species, lacking conserved motifs. CKS genes are broadly conserved, except in the charophyte algae *Mesostigma viridae* (Fig. 1c). A recent analysis of CDK genes in Marchantia identified a similar number of CDKs (Chen et al. 2025). Some were wrongly assigned to the CDKA clade instead of CDKAB. An additional CDKG (Mp2g21120) was also identified (Chen et al. 2025).

Downstream of CDK signaling, the RBR pathway is essential for cell cycle progression and is also highly conserved, with a single RBR homolog found in most species. There are 3 clades of RBR-related TFs that form part of the DREAM complex during the G1/S transition (E2F, DP, and DEL). The 3 clades share a common evolutionary origin (Rauber et al. 2016). E2F and DP TFs form highly conserved heterodimers to regulate the onset of DNA replication. Both protein families are present in animals and plants, suggesting an early origin for the interaction (Fig. 1c). In contrast, DEL proteins, which are present in both brown algae and plants, have been lost in several plant lineages.

The 3R-MYB TFs (c-Myb in animals), which regulate the G2/M transition (Ito et al. 2001), show a common origin before the divergence of plants and animals (Kobayashi et al. 2015; Feng et al. 2017), with some lineages losing 1 of the MYB domains. All plant species contain at least 1 3R-MYB gene, except for the algae species *Chlamydomonas* and *Penium margaritaceum* (Fig. 1c).

We found members of CDC20 and CDH1components in all species analyzed (Fig. 1c), except for *Prasinoderma coloniae*, which possess only 1 copy that is ancestral to both subfamilies (Dataset S1). This highlights the ubiquitous conservation of the APC/C complex across eukaryotes. Interestingly, in nonseed plants there is an additional copy of the CDC20 complex (Fig. 1c).

Among CDK repressors, KRP are found in both plants and animals with a very scattered presence among unicellular eukaryotes, involving several lineage-specific losses (Fig. 1c). The KRP family is expanded in vascular plants, but it contains a single member in bryophytes. On the other hand, WEE is found across eukaryotes, usually as a single member. Interestingly, we found a second clade of WEE that is present only in bryophytes and some streptophyte algae. The well-known family of dual-specificity phosphatases CDC25 is critical for the regulation of the cell cycle in metazoans, but no true homolog was identified in land plants so far. A functional copy of this component was identified in the green algae *Ostreococcus tauri* (Khadaroo et al. 2004) but not in the model *C. reinhartii*. This missing component of the canonical eukaryote cell cycle has puzzled cell cycle scientists for decades (Boudolf et al. 2006). In our analysis, we found that there are clearly 2 clades of CDC25 with at least 1 copy in most streptophytes. While the clade conserved in Arabidopsis and other vascular plants is specific from streptophytes, the other clade is homologous to the canonical CDC25 well characterized in metazoan. This means that this component was lost in vascular plants, as well as other lineage-specific losses (Fig. 1c, Dataset S1). In charophytes, including the algal sister lineage of land plants (Feng et al. 2024), green algae (Robbens et al. 2005), and bryophytes, most cell cycle genes exist as single copies, suggesting that the common ancestor of land plants possessed a minimal set of these core components. This set has expanded considerably in some lineages, particularly in flowering plants, consistent with the pattern of whole-genome duplications. Remarkably, the model liverwort *Marchantia polymorpha* retains a simplified set of cell cycle regulators, with apparently reduced redundancy compared with vascular plants. It has single copies of CYCA, RBR, E2F, CDKA, and most CDKs. Only some components have multiple copies, including CYCD (Mp*CYCD;1* and Mp*CYCD;2*), CYCB (Mp*CYCB;1* and Mp*CYCB;2*), CDKC (Mp*CDKC;1* and Mp*CDKC;2*), CDC20 (Mp*CDC20;1*, Mp*CDC20;2*), and 3 DP (Mp*DP1*, Mp*DP2*, and Mp*DP3*), 2 DEL (Mp*DEL1* and Mp*DEL2*), and 9 3R-MYBs (Mp*3R-MYB1* to Mp*3R-MYB9*).

### Expression profiles of cell cycle genes in Marchantia during development

To investigate the dynamic expression of key cell cycle regulators in Marchantia, we reanalyzed RNA-seq time-series data from 2 natural biological processes that feature a semi-synchronized reentry to the cell cycle: meristem regeneration (Ishida et al. 2022) and sporeling germination (Bowman et al. 2017). During regeneration, cells begin proliferating approximately 16 h after meristem excision (Nishihama et al. 2015). Similarly, dormant spores start dividing between 29 and 48 h after exposure to light (Attrill et al. 2024).

**Figure 2:**
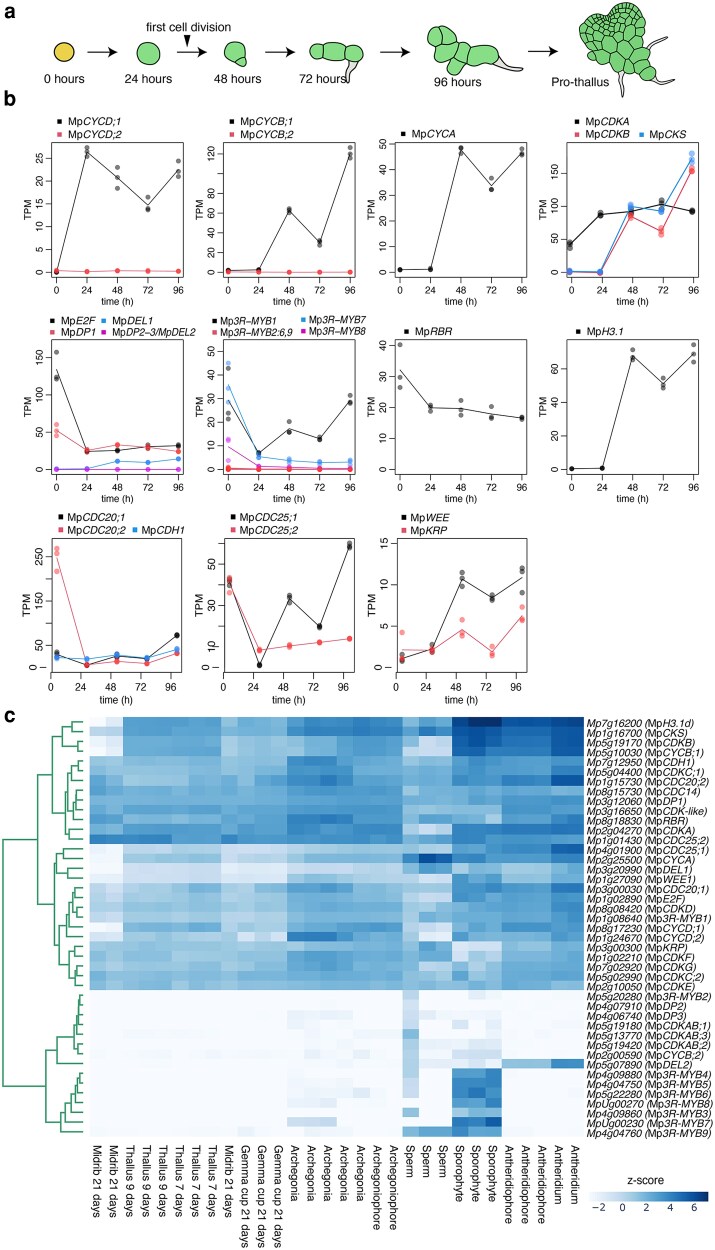
Expression of core cell cycle genes in Marchantia during development. a) Diagram of sporeling germination. b) RNA-seq analysis of representative cell cycle genes during the time course of sporeling germination (Bowman et al. 2017). Genes are grouped per family and distinguished by color (see legend). Individual points represent biological replicates and lines averages. c) Clustergram of cell cycle genes across Marchantia tissues during its life cycle as indicated in the *x* axis. Colors represent Z-scores of normalized TPM. Abbreviation: TPM, transcripts per million.

During sporeling germination, Mp*CYCD;1* transcript accumulated after 24 h of light exposure, before the first cell division, followed by peaks in Mp*CYCB;1*, Mp*CYCA*, Mp*CDKB*, and Mp*CKS* at 48 h (Fig. 2a). We also employed *HISTONE H3.1* (Mp*H3.1*) transcript accumulation as a reference marker for DNA replication, which also peaked 48 h after light exposure. The same was observed for other canonical components of the cell cycle, such as Mp*CDC25;1*, Mp*CDC20;1*, and Mp*WEE*. On the other hand, Mp*CDC20;2*, Mp*CDC25;1*, and Mp*CDC25;2* accumulate in dormant spores A similar pattern was observed during regeneration (Fig. S1), consistent with previous reports (Nishihama et al. 2015). These findings are consistent with a role for Mp*CYCD;1* in G1, driving cell cycle reentry. On the other hand, Mp*CDKA*, other CDKs, Mp*RBR,* Mp*KRP*, Mp*CDH1*, and Mp*CYCD;2* are constitutively expressed (Fig. 2b and c, Figs. S1 and S2). Mp*E2F* and Mp*DP1* transcription factors showed moderate but detectable upregulation (Fig. 2b).

Some predicted cell cycle genes showed low or no detectable expression during both regeneration and sporeling germination, including: Mp*CYCB;2*, *MpCDKAB;1-3*, and Mp*DP2 MpDP3* (Fig. 2b, Fig. S1). Mp*3R-MYB1* remained the most abundantly expressed *3R-MYB* ortholog in the vegetative gametophyte. By contrast, Mp*3R-MYB3* to Mp*3R-MYB9* were specifically expressed during early sporophyte development, and Mp*3R-MYB7* and Mp*3R-MYB8* were detected at the spore stage too (Fig. 2c). These 3R-MYB homologs belong to a different clade compared with 3R-MYB1 expressed in the gametophyte (Fig. 2c). Something analogous was observed with Mp*DEL2*, which is expressed in the antheridium (Fig. 2c). These data suggest that in the vegetative gametophyte, a simplified cell cycle regulation network is active, with only 1 copy of each transcription regulator, while some specialization could be expected in the sporophyte and during gametogenesis.

### Cell cycle progression shows phase-specific transcriptional dynamics

**Figure 3:**
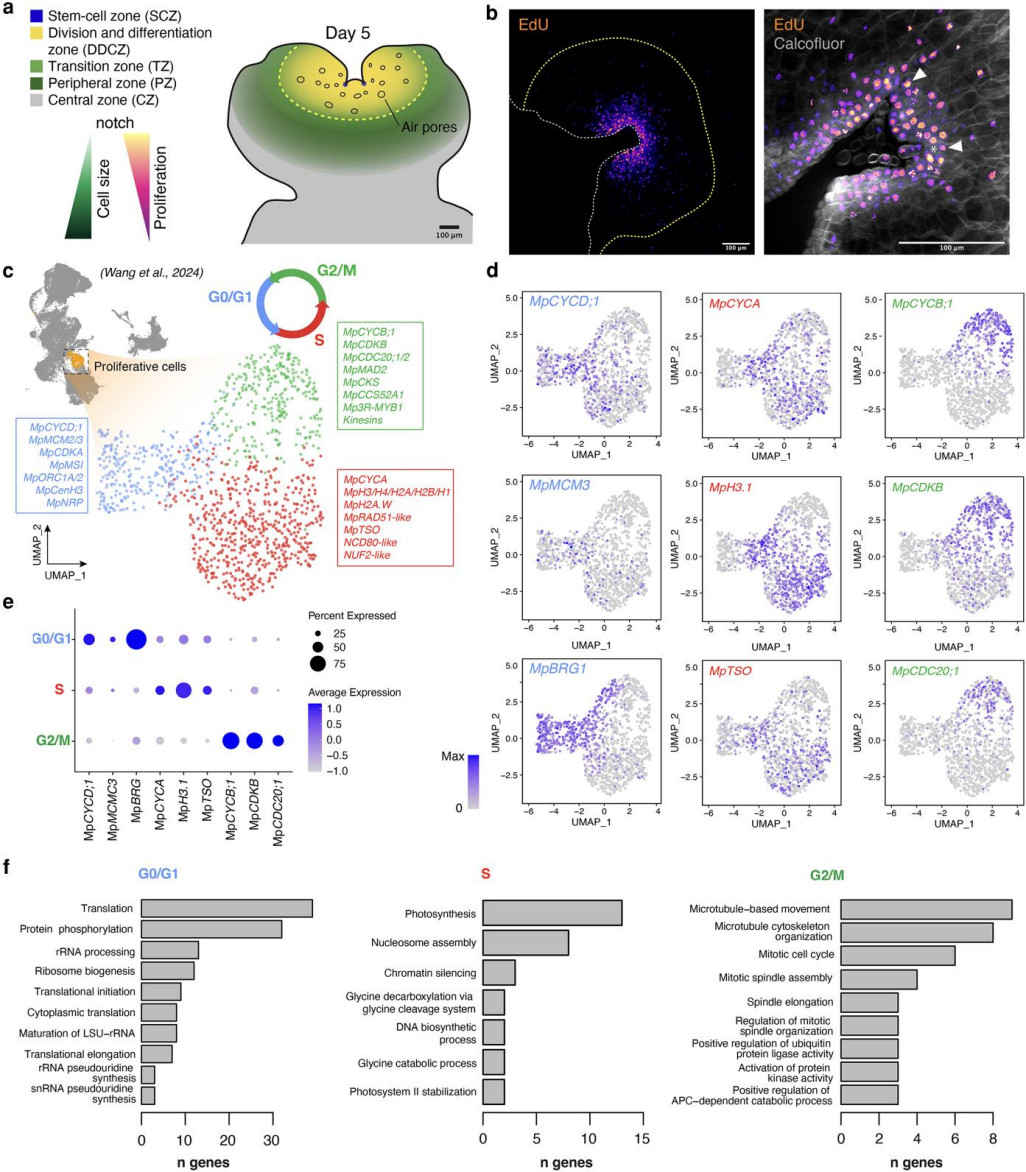
Spatio-temporal patterns of gene expression during cell proliferation. a) Diagram of cellular domains associated with cell proliferation in the Marchantia gemmaling b) Left, Confocal maximum intensity projection of EdU labeling in the Marchantia meristem in 5-d-old gemmalings. The white dashed line delimits the outline of the plant. The yellow dashed line represents the boundary of the mature epidermis. Arrow head indicates apical cells. Scale bars represent 100 µm. Right, optical clearing of the same plant focused on the apex. Arrows highlight apical cells. c-e) Classification of cells within the proliferative zone of the Marchantia gemma as shown in the legend. A UMAP based on scRNA data for all cells in the gemma is shown in the top left corner with proliferative cells highlighted in orange c). UMAP of the proliferative cells and classification into 3 subclustering (G0/G1, light blue; S, red; and G2/M, green). Cell cycle-related genes for each cluster are highlighted in colored square boxes. Feature plots d) and dot plot e) of representative genes. f) Top GO enrichment terms in their respective sub-clusters. Abbreviations: *MCM (MINICHROMOSOME MAINTENANCE*), *CDC* (*CELL DIVISION CYCLE*), *MSI* (*MUSASHI*)*, ORC* (*ORIGIN RECONGNITION COMPLEX*), *CCS52A* (*CELL CYCLE SWITCH PROTEIN 52 A*), *NDC* (*NUCLEAR DIVISION CYCLE*), *NUF* (*KINETOCHORE PROTEIN NUF*), *BRG* (*BLEOMYCIN RESISTANT GENE*), *NRP* (*NAP-RELATED PROTEIN*). Abbreviation: TPM, transcripts per million.

To further investigate cell divisions and growth in planta, we focused on the early stages of Marchantia gemma development. The meristem undergoes a transition from an immature state to the fully mature state around 5 d post germination (Romani et al. 2024). We performed 5-ethynyl-2′-deoxyuridine (EdU) labeling to map actively dividing cells. As expected, EdU-positive cells were concentrated around the division and differentiation zones (DDCZ) and the stem-cell zone (SCZ), including the apical cell (Fig. 3a and b). This apical notch region is the main focal point for vegetative growth in Marchantia where most cell divisions take place. Cells near the apical notch proliferate and differentiate, forming specialized cells and air pore structures. Epidermal cells exit the cell cycle and elongate as they become distal to the apical notch (Fig. 3a and b).

To elucidate the expression of cell cycle regulators during gemmaling development, we generated transcriptional reporters using Golden-Gate compatible constructs (Sauret-Gueto et al. 2020; Romani et al. 2024). Transcriptional reporters showed that the key components were actively expressed in the EdU-positive zone (Fig. S3), corresponding to the DDCZ of the vegetative meristem. Consistent with the transcriptomic analysis, the promoter of Mp*CYCB;2* showed no detectable expression, suggesting it could be a pseudogene. *_pro_*Mp*CYCD;2* exhibited a more irregular pattern compared with *_pro_*Mp*CYCD;1*, which was consistently expressed in dividing cells. Mp*3R-MYB1* and Mp*E2F* are also actively expressed in dividing cells, consistent with prior transcriptomic analyses in the gametophyte (Fig. S3). Expression of DP TFs reporters was not detectable at this stage (Romani et al. 2024). This is inconsistent with Mp*DP1* transcriptomics, likely due to the promoter being insufficient to capture all regulatory elements important for this gene.

To gain a more refined understanding of the dynamic expression of cyclins during all phases of the cell cycle, we leveraged single-cell RNA-seq data obtained from growing gemmalings. Previous work identified clusters of cells proliferating around the apical notch, including cell cycle related genes (Wang et al. 2023). Subclustering of this group revealed 3 clearly distinct transcriptional profiles (Fig. 3c).

Using well-characterized cell cycle markers, we found that these subclusters correspond to G0/G1, S, and G2/M phases. The G0/G1 subcluster was characterized by the accumulation of Mp*CYCD;1* and Mp*MCM3*, homologs of *Arabidopsis* G1-phase markers (Zhang et al. 2021), and other transcripts associated G1, cyclin degradation, and initiation of replication (Fig. 3c–e, Table S1). The S-phase cluster was defined by replication-related genes, including Mp*CYCA*, several histones, and DNA damage repair genes such as Mp*RAD51* (Kutashev et al. 2024). The G2/M subcluster showed elevated transcription of mitotic markers, including the expected cyclin CDKs: MpCYCB;1, MpCDKB, MpCKS (Lee et al. 2024), and other typical G2/M components such as microtubule components (tubulins and kinesins) and Mp*3R-MYB1*,Mp*CCS52A1/CDH1*, MpAPC11, and both *CDC;20* coactivators (Mp*CDC20;1* and Mp*CDC20;2*) (Fig. 2e and f). Interestingly, the CDC25 homologous to the canonical CDC25 from yeast (MpCDC25;1) was G2/M phase specific, while the second copy (MpCDC25;2), homologous to the Arabidopsis CDC25, was ubiquitously expressed. Among the other CDKs, MpCDKA showed differential transcription in the G0/G1 cluster, Mp*CDKD* in the G2/M cluster and Mp*CDKG* and Mp*CDK-like* in G0/G1, comparable with their homologs in *Arabidopsis* (Menges et al. 2005). On the other hand, transcript levels of other key components of the cell cycle such as RBR and CKIs did not show a significant preferred accumulation in specific phases of the cell cycle (Table S1). In Marchantia, we found strong indications that cyclins could be used confidently as phase-specific markers, as expected from other eukaryotes. This contrasts with recent work in *Arabidopsis* (Lee et al. 2024; Vukasinovic et al. 2025), where cyclins were not reliable markers due to low levels of expression or redundancy. This is likely a consequence of the simplicity of Marchantia meristem and low redundancy of cell cycle genes.

This analysis also provided a comprehensive set of additional phase specific markers (Table S1). Using Gene Ontology (GO) enrichment analysis (Fig. 3f), we found that cell cycle–related terms were enriched in G2/M (eg “Microtubule-based movement” and “Mitotic cell cycle) and S phase clusters (eg “Nucleosome assembly” and “DNA biosynthetic process”). It is expected that G1 genes are not enriched in cell cycle associated with GO terms (Lee et al. 2024). A recent scRNA-seq analysis in Arabidopsis (Vukasinovic et al. 2025) obtained a comparable dataset in Arabidopsis to compare the degree of conservation. Among the genes with at least 1 eggNOG level (Huerta-Cepas et al. 2019; Cantalapiedra et al. 2021) in common with markers, 38% of the G0/G1 cluster in Marchantia was orthologous to the same group of markers in Arabidopsis, 63% of the S cluster, and 72% of the G2/M. While some overlap between G0/G1 and S phase could be expected, probably due to methodological differences, the G2/M cluster is very similar between both analyses. Overall, this suggests a very high conservation between phase specific genes in Marchantia.

### Spatiotemporal control of cyclins and cyclin-dependent kinases in Marchantia

**Figure 4:**
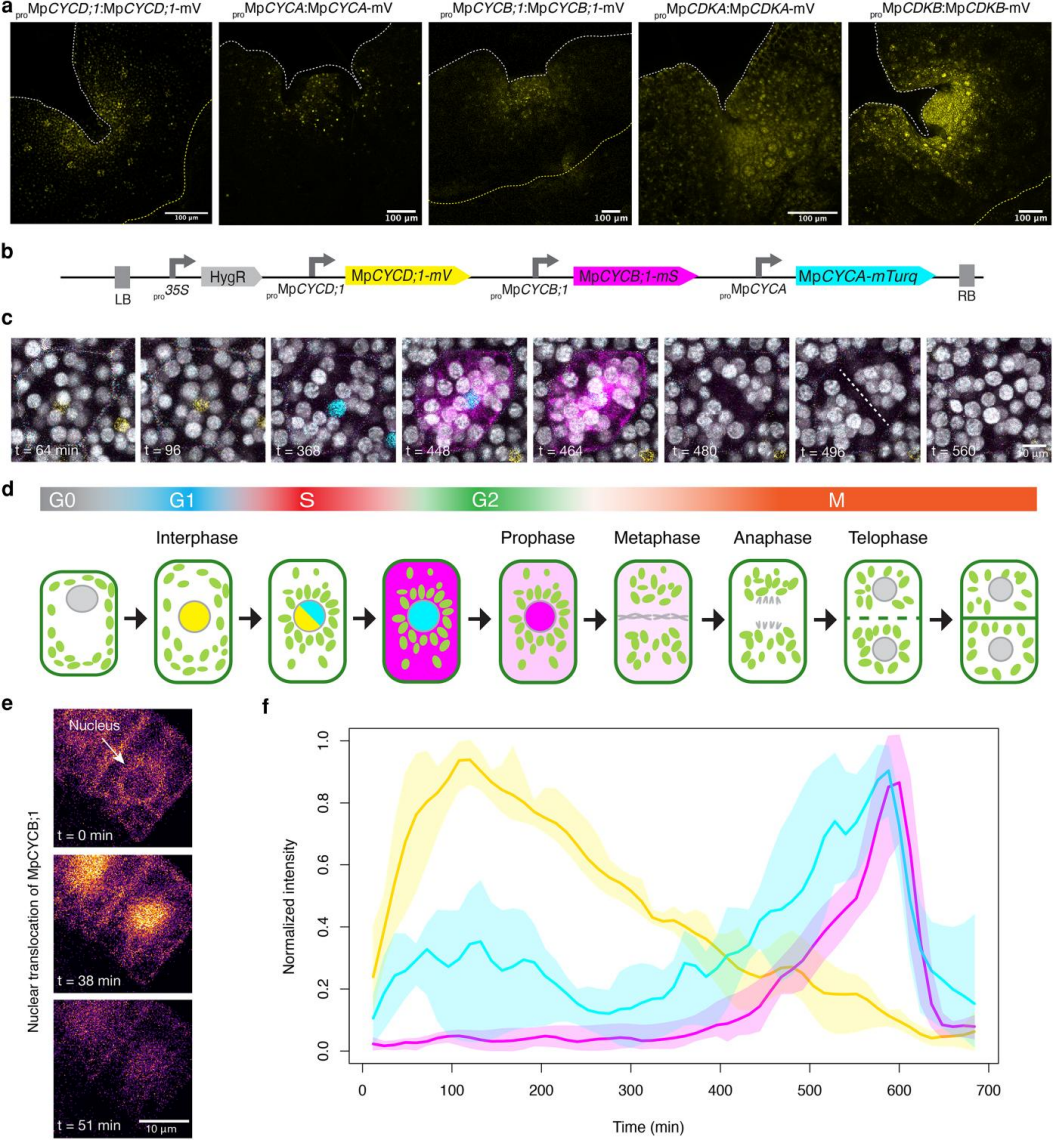
In vivo expression of cyclins and CDKs in Marchantia. a) Expression of cyclin and CDKs translational reporters (yellow) in 5-d-old gemmalings. Images are representative of 4 independent lines obtained per construct. The white dashed line delimitates the outline of the plant. The yellow dashed line represents the boundary of the mature epidermis. b) Diagram of gene construct combining 3 translational reporters for the main cyclins in Marchantia. c) Time-lapse of MpCYCD;1 (yellow) MpCYCA (cyan), and MpCYCB;1 (magenta) translational reporters of an individual cell dividing during regeneration. Chlorophyll (gray) is shown to visualize the cell division phases using chloroplast movement. Dashed lines indicate the new cell division plane. d) Schematic representation of cyclin expression and subcellular localization dynamics during cell division. e) The translocation of MpCYCB;1 (inferno LUT) from the cytosol to the nuclei at 2 different frames. f) Quantification of normalized fluorescence signal over a cell division (n = 3) for the triple reporter. Scale bars are indicated in each panel.

Cyclins are not only subjected to tight transcriptional control during cell division but also protein degradation. We made translational reporters (for MpCYCD;1/2, MpCYCB;1, MpCYCA, and MpCDKA) to better capture the dynamics of protein accumulation during cell division. Each reporter includes the native promoter and 5′UTR driving the expression of the full CDS sequence fused to a C-terminal mVenus (mV) fluorescent protein. Compared with promoter fusions for the corresponding genes, the signals were more restricted to dividing cells. MpCYCD;1 was highly expressed in dividing cells, as expected, and rhizoid precursors and air pores (Fig. 4a, Fig. S4a). On the other hand, MpCYCD;2 translational reporter was undetectable in vegetative tissues. MpCYCB;1 has a typical expression pattern “salt and pepper” expression pattern, with scattered cells, (Fig. 4a) as described for its homologs in flowering plants (Colon-Carmona et al. 1999). A similar pattern was observed for MpCYCA (Fig. 4a). The MpCDKA translational reporter is broadly expressed across the mature thallus and not restricted to dividing cells (Fig. S5a). Finally, MpCDKB is more restricted to dividing cells (Fig. S5b).

For time-lapse imaging, we took advantage of cell cycle reentry that is triggered during regeneration. This allows a clear visualization of cellular features along with expression dynamics during cell division using the translational reporters. Live imaging showed that MpCYCD;1 protein accumulated in the nuclei of cells before cell division and was rapidly degraded before the start of the prophase (Fig. 4a, Video S1). Similarly, MpCYCA also accumulated in the nuclei until prophase and was then rapidly degraded (Fig. 4a, Video S2). Finally, MpCYCB;1 was transiently expressed in the cytosol and accumulated during metaphase/anaphase (Fig. 4a, Video S3).

Fluorescent cell cycle indicators are powerful tools to understand the spatiotemporal control of cell proliferation (Sakaue-Sawano et al. 2008; Desvoyes et al. 2020) but often rely on downstream signals and not cyclins themselves. The simplicity of cyclins in *Marchantia* provides an opportunity to coexpress them simultaneously in individual cells and directly visualize the relative timing and patterning of expression suggested by scRNA-seq and individual translational fusions. We generated a reporter line expressing the 3 predicted main cyclins under their native promoters in the same plasmid fused to different fluorescent proteins (Fig. 4b) and performed live imaging on dividing cells. As expected, expression of MpCYCD;1 was observed in cells that are going to divide, followed by MpCYCA, and finally MpCYCB;1 as predicted before (Fig. 4c and d). Intriguingly, MpCYCB;1 was relocalized to nuclei before its full degradation after the dismantling of the nuclear envelope (Fig. 4e). The degradation of MpCYCA occurred immediately after MpCYCB;1 was translocated to the nucleus. MpCYCB;1 expression persisted shortly after nuclear envelope disassembly and mitosis took place (Fig. 4c and d). We quantified the relative signal of each of the cyclins in single nuclei during cell division, giving us a detailed and ordered series (Fig. 4f). Overall, this highlights the importance of protein degradation and subcellular localization controlling cyclin-dependent activity. These results align with the respective roles of the predicted main cyclins and experimentally verified markers identified in scRNA-seq experiments.

We also looked at translational reporters for MpCDKA and MpCDKB. Both were localized in the cytosol and nucleus, and fluorescence was visible throughout the cell cycle. While MpCDKA strongly accumulated in the interphase (Fig. S6d, Video S4), MpCDKB was found to be more prominent later during mitosis (Fig. S6b, Video S5).

The molecular sequences of cyclin and CDK components showed a high similarity with their orthologues in plants and animals. The main difference is that the combination of potential pairings is quite reduced compared with the complex network in *Arabidopsis* (Boruc et al. 2010a). It is expected that protein-protein interactions will be conserved too. To test this, we first simulated the protein-protein interactions between all cyclins and their CDK pairs using AlphaFold3 (Abramson et al. 2024). All cyclins showed a high-confidence ipTM score (>0.8) for interactions between MpCDKA and MpCDKB, except for MpCYCD;2 and both CDKs and MpCYCD;1-MpCDKB, which fall in the medium-confidence range, and relatively lower scores for combinations involving other CDKs (Fig. S7a and b). To confirm this in planta, we performed bimolecular fluorescent complementation (BiFC) in Marchantia sporelings. We cloned the N terminal and C terminal of YFP to the CDS of cyclins and CDKs, respectively, with different selectable markers (either hygromycin or chlorsulfuron) and analyzed the reconstitution of YFP in transgenic lines for the different combinations of pairs. We found that MpCYCD;1 and MpCYCA can interact with MpCDKA and MpCDKB in the nucleus, while MpCYCB;1 also interacts with both in the cytosol as well as the nucleus (Fig. S7c). Some protein-protein interactions between cell cycle components were also recently studied by Chen et al. (2025) by bioinformatic analyses and yeast 2-hybrid (Y2H) assay. Among them, the confirmation of the interaction with MpCKS with MpCDKA and MpCDKB (Chen et al. 2025) by Y2H are the most valuable in the context of our work. Overall, these results align with the conserved roles of the main cyclins and CDKs in Marchantia in the progression of the cell cycle. Interestingly, we did not observe any specificity between different pairs at the protein-protein interaction level.

### Distinct roles of Marchantia cyclins revealed by overexpression analyses

**Figure 5:**
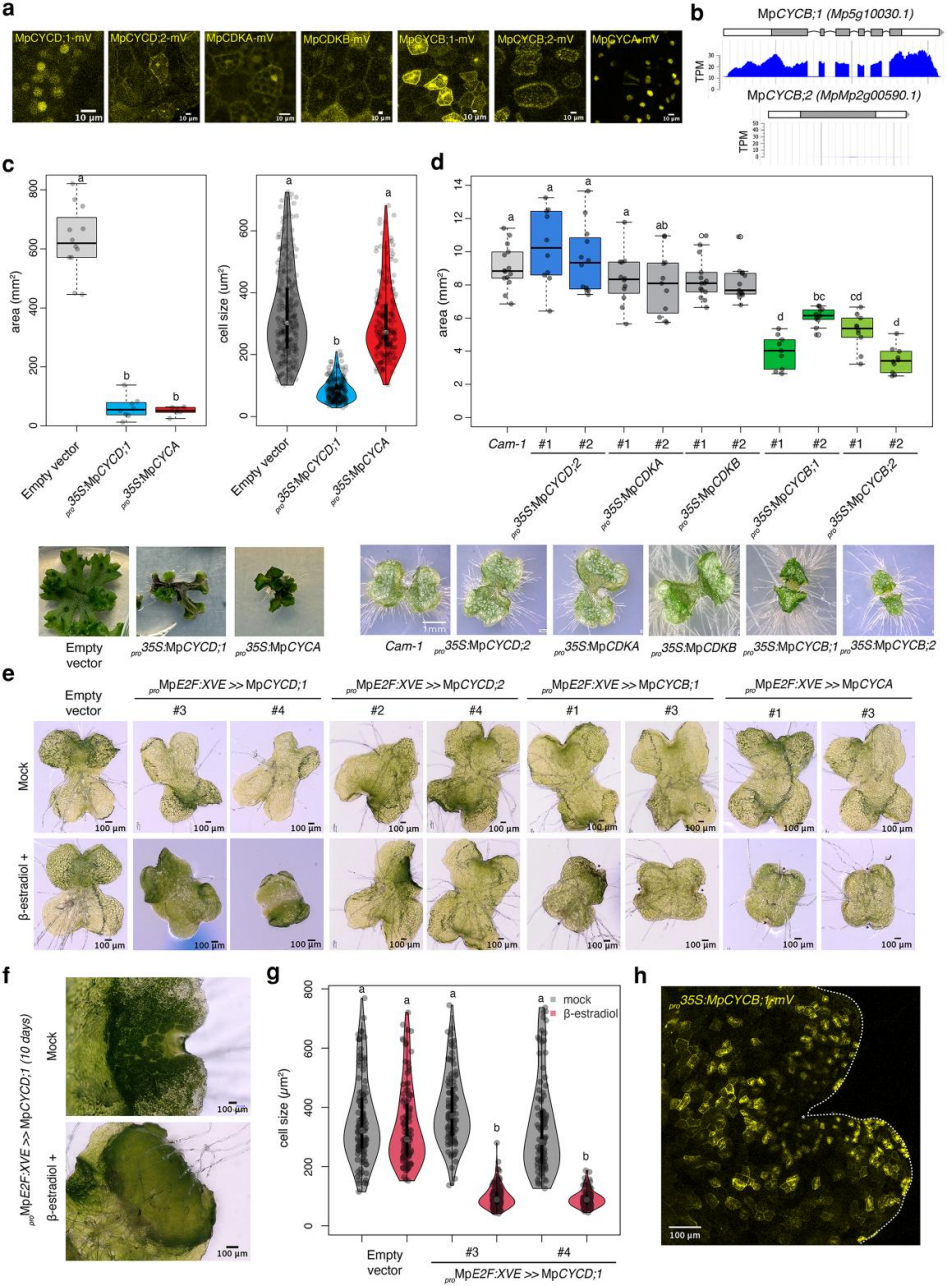
Functional characterization of cyclins and CDKA. a) Subcellular localization of selected cyclins and CDKA protein fusions with mVenus. b) Overview of the genomic locus of Mp*CYCB;1* and Mp*CYCB;2*, including a diagram of intron structure and average RNA-seq coverage in mixed tissues. c) Boxplot of plant area (left) and cell size (right) of *_pro_35S:*Mp*CYCD;1-mVenus* primary transformant plants. Representative images of the plants are shown beneath the plots. d) Boxplot of plant area of *_pro_35S:*Mp*CYCD;2-mVenus*, *_pro_35S:*Mp*CDKA-mVenus*, *_pro_35S:*Mp*CYCB;1-mVenus*, and *_pro_35S:*Mp*CYCB;2-mVenus* constitutive overexpression in 7-d-old gemmalings. Representative images of the plants are shown at the bottom. e) Representative images of gemmalings of transgenic plants with inducible overexpression of cyclins grown for 3 d in 0.5 × Gamborg B5 supplemented with 5 μM β-estradiol or DMSO (mock). Transgenic constructs are indicated in the images. f) Same for 10-d-old gemmalings of Mp*CYCD;1.* g) Violin plots of cell size of inducible Mp*CYCD;1* (*_pro_*Mp*E2F:XVE >>* Mp*CYCD;1*) in 3-d-old plants. h) Confocal microscopy image of *_pro_35S:*Mp*CYCB;1-mVenus* (yellow) overexpression. The white dashed line delimitates the outline of the plant. The yellow dashed line represents the boundary of the mature epidermis. Control indicates a transgenic line transformed with an empty vector. Scale bar lengths are indicated in the images. Symbols above the bars indicate grouping by *P*-value < 0.001 in a Tukey honest significant difference method.

To study the role of cyclins and their functional conservation, we generated fluorescent protein fusions (CDS-mVenus) for overexpression of cyclins and CDKA/B. MpCYCD;1 is localized in the nucleus as are most D-type cyclins in *Arabidopsis*, while MpCYCD;2 showed a broader distribution in the cytosol (Fig. 5a). As shown before, CDKA and CDKB are cytosolic (Fig. 5a), as is its ortholog in *Arabidopsis* (Boruc et al. 2010b). Constitutive overexpression of Mp*CYCD;1* or Mp*CYCA* resulted in smaller plants that did not produce gemma cups and showed severe developmental defects (Fig. 5c). Plants overexpressing MpCYCD;1 also showed reduced cell size (Fig. 5c). In contrast, overexpression of Mp*CYCD;2*, Mp*CDKA*, and *MpCDKB* had no noticeable effects on plant growth under the conditions tested (Fig. 5d).

Mp*CYCB;1* and Mp*CYCB;2* share high sequence similarity (90.1%), but Mp*CYCB;2* lacks the conserved intron structure, and its endogenous expression is undetectable (Fig. 2, Fig. S1, Fig. 5b). Both B-type cyclins localize to the cytosol and nucleus (Fig. 5a), consistent with the homologs in *Arabidopsis* (Boruc et al. 2010b). Overexpression of either Mp*CYCB;1* or Mp*CYCB;2* impaired growth, resulting in smaller plants (Fig. 5d). This indicates that despite signs of pseudogenization, Mp*CYCB;2* encodes for a functional protein. Imaging of plants overexpressing MpCYCB;1 indicated that cells with higher mVenus signal were arrested in mitosis (Fig. 5h, Video S6); this contrast with the transient expression was observed when it was under the native promoter control (Fig. 4a, Video S7).

We also generated lines with inducible expression of Mp*CYCD;1*, Mp*CYCD;2*, Mp*CYCB;1*, and Mp*CYCA* using an β-estradiol XVE system (Siligato et al. 2016) under the control of _pro_MpE2F (Ishida et al. 2022). Inducible overexpression of MpCYCD;1 was sufficient to trigger the reentry into the cell cycle, producing ectopic cell division and overproliferation. We observed accumulation of significant numbers of smaller, undifferentiated cells after MpCYCD;1 induction (Fig. 5g). This is consistent with its canonical role for D-type cyclins in initiating cell cycle progression (Fig. 5f and g). In similar experiments, induction of Mp*CYCD;2* expression did not cause any observed developmental defect. On the other hand, inducible overexpression of Mp*CYCB;1* or Mp*CYCA* caused strong cell cycle arrest (Fig. 5e and h, Video S6). Unlike MpCYCB;1, in MpCYCA overexpression, most of the fluorescence signals are nuclear as the nuclear envelope remained intact (Fig. 5a). Overall, this supports the key roles of Mp*CYCD;1*, Mp*CYCB;1*, and Mp*CYCA* in cell cycle control.

**Figure 6:**
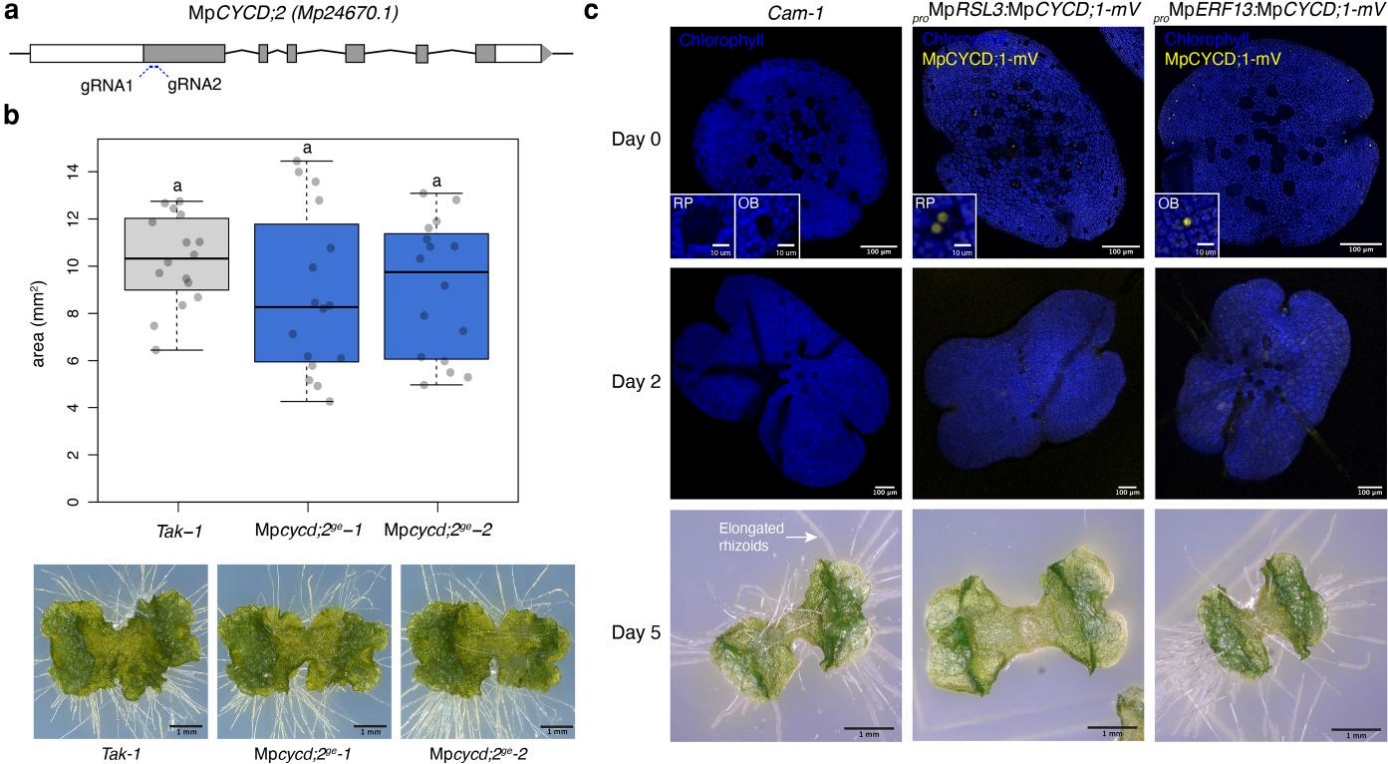
Mp*CYCD;2* knockout and cell-type specific overexpression of Mp*CYCD;1* using cell type–specific promoters. a) Structure of MpCYCD;2 locus with the position of designed guide RNA (gRNA). Exons are shown as boxes. b) Boxplot of plant area of 7-d-old gemmalings Mp*cycd;2^ge^* and wild-type plants (*Tak-1*). Representative images of the plants are shown at the bottom. Scale bar lengths are indicated in the images. Symbols above the bars indicate grouping by *P*-value < 0.001 in a Tukey honest significant difference method. c) Plants expressing *_pro_*Mp*RSL3:*Mp*CYCD;1-mVenus* (rhizoid precursor specific) and *_pro_*Mp*ERF13:*Mp*CYCD;1-mVenus* (oil body specific). Wild-type Cam-1 was used as a control. Confocal full-stack images of representative individual gemmalings (0 and 3 d old) are shown with mVenus (yellow) and chlorophyll (blue) channels merged. The mVenus channel is not shown in the wild type. Representative picture of 5-d-old gemmalings. Scale bar lengths are indicated in the images. Abbreviations: OB, oil bodies; RP, rhizoid precursors.

To examine whether Mp*CYCD;2* plays any role in proper vegetative development in Marchantia, we generated CRISPR-Cas9 knockout lines. We obtained independent lines using 2 different gRNAs, generating 20-bp (Mp*cycd;2-1^ge^*) or 1-bp (Mp*cycd;2-2^ge^*) deletions (Fig. 6a, Fig. S8) that caused frame shift and early stop codons. We did not observe any significant defect in growth of the lines (Fig. 6b) compared with wild-type plants. Considering the results of overexpression, this suggests that Mp*CYCD;2* does not affect cell proliferation during vegetative growth.

### Cell proliferation interferes with differentiation

Morphological defects observed after Mp*CYCD;1* overexpression indicated that cell differentiation was disrupted when cell division was ectopically induced (Fig. 5c and f). To explore this interaction, we employed cell type–specific promoters to target Mp*CYCD;1* misexpression to differentiating cells, specifically rhizoid precursors and oil body cells (Sauret-Gueto et al. 2020; Romani et al. 2024). Both cell types, identifiable by their reduced chlorophyll content and distinct morphology, showed specific ectopic expression of Mp*CYCD;1* at the gemma stage. Misexpression of Mp*CYCD;1* under a specific rhizoid promoter (*_pro_MpRSL3*) caused ectopic cell divisions, resulting in the suppression of rhizoid development (Fig. 6c). Similarly, misexpression in oil body cells (*_pro_MpERF13*) triggered ectopic divisions, leading to smaller oil body cells in the gemma (Fig. 6). Notably, these effects were not observed in Mp*CYCB;1* misexpression lines (Fig. S9).

During regeneration, both rhizoid precursors and oil body cells can undergo division and regenerate an entire plant after cell reprogramming (Nagai 1919; Romani et al. 2024). De-differentiation of rhizoid precursors into epidermal cells is a normal phenomenon in some dorsal but not in ventral rhizoid precursors (Romani et al. 2024). The misexpression of Mp*CYCD;1* appears to extend this phenomenon, promoting widespread de-differentiation across ventral and dorsal precursors, impacting the emergence of rhizoids.

### MpKRP plays a conserved function as cell cycle inhibitor

So far, we established the expression and putative roles of key proteins in cell cycle progression in Marchantia. Several other regulators of cell cycle machinery could be explored. For example, it has been shown that overexpression of CKS or WEE causes an increased cell cycle duration and growth arrest in Arabidopsis (De Veylder et al. 2001a; De Schutter et al. 2007). Similarly, in Marchantia, we showed that the overexpression of either MpCKS or MpWEE caused strong growth arrest (Fig. S10).

**Figure 7:**
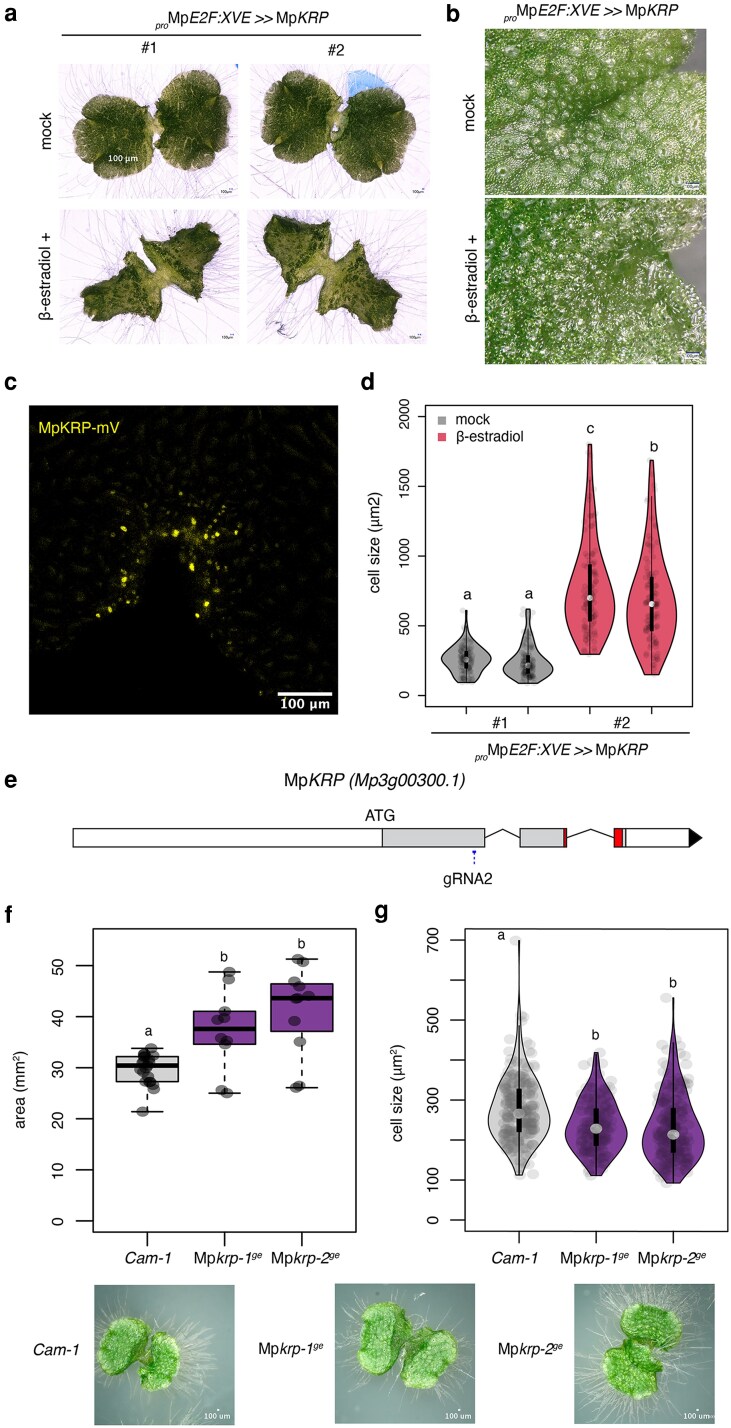
Functional characterization of Mp*KRP*. a) Representative images of gemmalings of transgenic plants with inducible overexpression of cyclins grown for 7 d in 0.5× Gamborg B5 supplemented with 5 μM β-estradiol or DMSO (mock). Transgenic constructs are indicated in the images. b) Closer look to the apical region. c) Confocal microscopy image of *_pro_*Mp*E2F:XVE >>* Mp*KRP* (yellow) overexpression in 4-d-old gemmalings supplemented with 5 μM β-estradiol represents the boundary of the mature epidermis. d) Violin plots of cell size of *_pro_*Mp*E2F:XVE >>* Mp*KRP* in 3-d-old plants. e) Structure of Mp*KRP* locus with the position of designed guide RNA (gRNA). Exons are shown as boxes. f-g) Boxplot of plant area f) and violin plot of cell size g) of 7-days old gemmalings Mp*krp^ge^* and wild-type plants (Cam-1). Representative images of the plants are shown at the bottom. Scale bar lengths are indicated in the images. Symbols above the bars indicate grouping by *P*-value < 0.001 in a Tukey honest significant difference method.

The roles of KRP inhibitors could be interesting in the context of the lack of endoreduplication in Marchantia (Nishihama et al. 2015). Inducible overexpression of Mp*KRP* significantly inhibited cell division and promoted cell expansion, resulting in the formation of large cells up to ∼3 times normal size (Fig. 7a–d), suggesting that the KRPs play a conserved role in cell cycle inhibition. MpKRP is localized to nucleus (Fig. 7c), as observed for their Arabidopsis homologs (Boruc et al. 2010b).

To examine their roles in a physiological context, we generated CRISPR-Cas9 knockout lines for MpKRP (*Mpkrp^ge^*). Mutant plants showed a slight but significant increase in plant area and smaller cell size compared with wild type (Fig. 7e–h; Fig. S8). No major morphological changes or defects in differentiation were observed, as rhizoids, air pores, and oil bodies remained unaffected.

## Discussion

Our comprehensive characterization of cell cycle regulation in Marchantia confirms the profound evolutionary conservation of eukaryotic cell division machinery across 2 billion years of evolution. The genetic simplicity of cell cycle control in this organism is stark. It is neither expanded (as in most land plants) nor seemingly reduced (as in yeasts). Marchantia possesses the most streamlined cell cycle machinery characterized by plants, with single functional copies of core cyclins and CDKs that typically exist as large gene families in flowering plants (Fig. 1). Comprehensive phylogenetic analysis of cell cycle components is an important tool for studying the evolution of these components in plants and beyond. It highlights the main elements conserved among plants and has the potential to bridge the gap in systems level understanding of cell division in unicellular algae and flowering plants.

**Figure 8:**
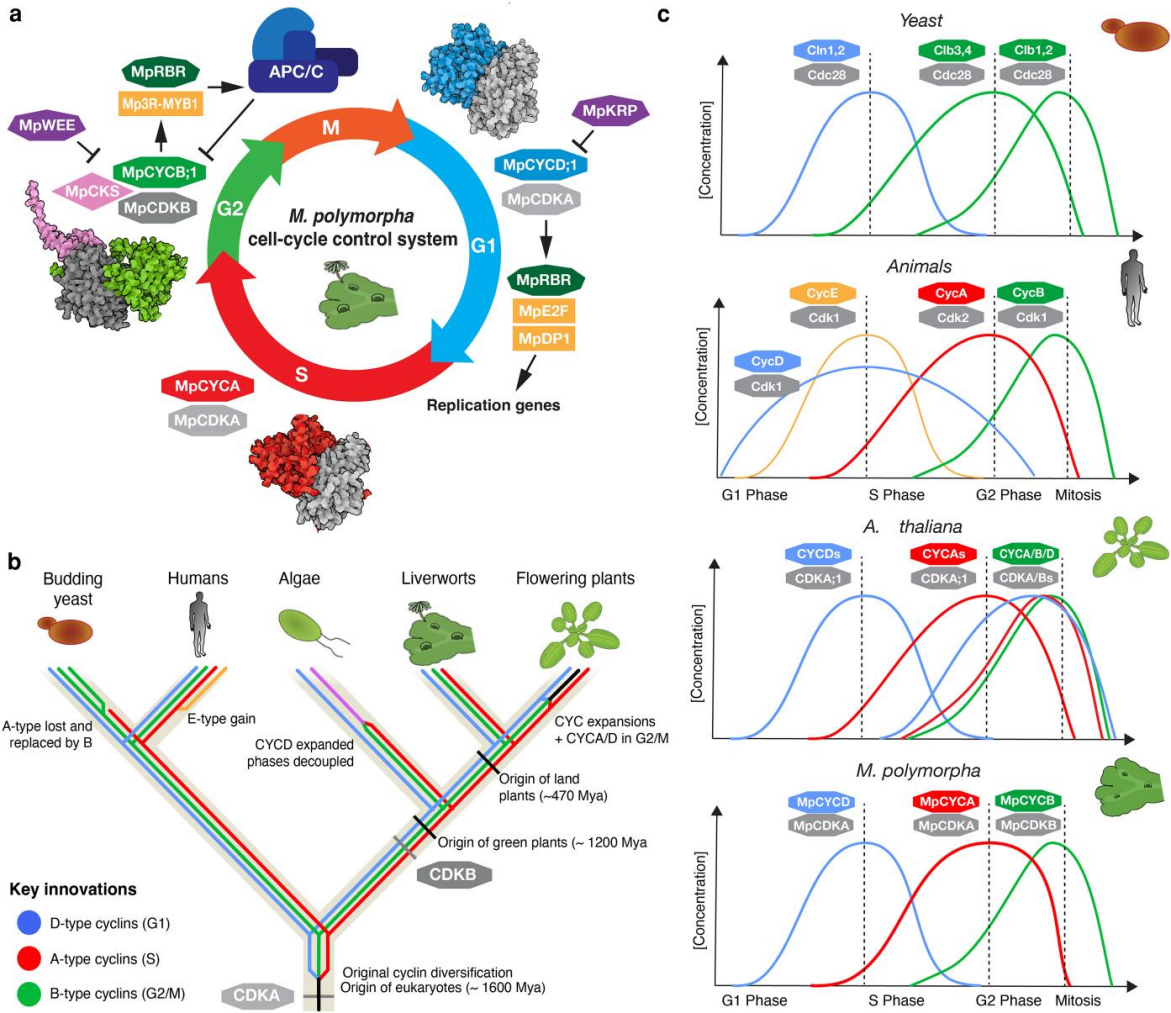
Core regulatory network of the cell cycle and phase specificity in Marchantia compared with other model species. a) Diagram of cell cycle control system in Marchantia. This diagram integrates expression and interactions experimentally verified in this work and hypothetical interactions inferred from conventional model systems. Protein complex structures were obtained using Alpha Fold 3 following the same color code. b) Phylogram of the evolutionary trajectory of cell cycle genes in model eukaryotes (*Saccharomyces cerevisiae*, *Homo sapiens*, *C. reinhartii*, *A. thaliana*, and *M. polymorpha*) and its possible ancestral state. c) Comparison of expression patterns of cyclins and CDKs.

The concordant phase assignments from scRNA-seq (Fig. 3c–f), single- and multi-cyclin translational reporters (Fig. 4a–f), and inducible perturbations (Fig. 5) collectively support a sequential, phase-ordered cyclin relay in the vegetative gametophyte. In particular, the expression of cyclins follows an archetypal pattern with each gene peaking at the corresponding phase of its putative function, resembling a sequence that can be traced back to animal and yeast models. As we successfully demonstrated using scRNA-seq and translational reporters in Marchantia, this sequential pattern of gene expression is characterized by MpCYCD;1 and MpCDKA during G1-phase, MpCYCA during S-phase, and MpCYCB;1 and MpCDKB in G2/M (Fig. 8a). The translational reporters generated here offer an unprecedented view of the cyclins relay in vivo and their protein dynamics. Ultimately, the phase specificity of the different components depends on a combination of factors, including transcriptional and post-transcriptional regulation and subcellular localization.

This also provides a compelling framework to understand cell division in plants, the formation of functional complexes in each phase, and their evolution. Based on phylogenetic analysis and functional studies in Marchantia and other species, it can be inferred that this simple configuration represents the ancestral state of cell cycle regulation in plants (Fig. 8b).

The streamlined system of cyclin-CDK components in Marchantia indicates that the more complex regulatory network is a derived feature in flowering plants. Thus, the extensive cyclin diversity observed in vascular plants likely arose through lineage-specific gene duplications, leading to redundancy and diversification. Subsequent neofunctionalization and subfunctionalization may have facilitated cellular innovations and tissue specificity. The set of key regulators that we describe here for Marchantia are expressed in both the haploid and diploid generation (Fig. S1). While all functional and single-cell analyses were performed in vegetative gametophyte, it is expected that a similar phase-ordered machinery operates across generations; future work in sporophyte and meiosis will test this prediction and define context-specific features.

The only 2 gene duplications among Class I cyclins in Marchantia, Mp*CYCB;2* is a putative pseudogene with negligible expression across development, while Mp*CYCD;2* belongs to a bryophyte-specific clade (CycD’). Following overexpression, subcellular localization, and examination of mutant phenotypes, we concluded that Mp*CYCD;2* is not a canonical D-type cyclin, and it is dispensable for vegetative growth. However, the precise role of this clade of cyclins in liverworts remains to be studied in more detail in other developmental stages. For example, MpCYCD;2 is strongly expressed in archegonia (Fig. 2c).

Establishment of a framework for the key cyclin-CDK components in a nonvascular plant like Marchantia provides an opportunity to re-examine their roles in an evolutionary context. Although CDKA represents the plant ortholog of the key eukaryotic CDK1, it is not strictly essential in *Arabidopsis*, *Physcomitrium*, and *Chlamydomonas* (Nowack et al. 2012; Tulin and Cross 2015; Bao et al. 2022). Instead, the plant-specific CDKB is necessary for spindle formation and nuclear division in *Chlamydomonas* (Tulin and Cross 2014; Pecani et al. 2022). In Marchantia, CDKA is steadily expressed in dividing cells, with some preference for G1, while MpCDKB is highly expressed at G2/M (Fig. 3). However, overexpression of either MpCDKA or MpCDKB did not appear to affect growth, and these might not be rate-limiting factors (Fig. 5). CDK translational reporters were detected throughout the cell cycle (Fig. 4) and did not exhibit a strict phase-specific posttranscriptional regulation as observed for cyclins.

D-type cyclins are conserved across eukaryotes and are undoubtedly important for plant growth and development, though their essentiality in plants remains unclear (Cross and Umen 2015). Overexpression of Mp*CYCD;1* in Marchantia produces phenotypes analogous to those observed for *CYCD3* homologs in flowering plants (Dewitte et al. 2003; Koroleva et al. 2004; Menges et al. 2006), reinforcing the idea that regulating cell cycle re-entry is the ancestral role of D-type cyclins in plants. This is important to understand the divergent roles of other D-type cyclins in flowering plants and contrast with the role of D-type cyclins in animals (Datar et al. 2000).

We also demonstrated a close relationship between cyclin-mediated cell cycle re-entry and cellular differentiation in Marchantia (Fig. 6). Our findings suggest that maintaining proper coordination of cell division rates is essential for preserving cell identity. This contrasts with Arabidopsis, where manipulating the cyclins in specialized cells, such as trichomes, can alter cell size and induce ectopic cell division but maintains cell identity (Schnittger et al. 2002a, 2002b). These differences may help explain Marchantia's remarkable regenerative capacity and developmental plasticity (Nishihama et al. 2015).

Interestingly, overexpression of Mp*CYCA* or Mp*CYCB;1* is deleterious in Marchantia, leading to cell cycle arrest. A-type cyclins play a critical role in cell division in *Chlamydomonas* but it is not essential (Atkins and Cross 2018). They are usually associated with ploidy level and meiosis (Imai et al. 2006; d’Erfurth et al. 2010). However, this might not be the case in Marchantia and other non-seed plants due to the absence of endoreduplication (Bainard et al. 2013; Nishihama et al. 2015). The absence of endoreduplication in Marchantia, unlike in seed plants, provides a simplified system to study mitotic cell cycle regulation without the complexity of alternative programs. Among the different A-type cyclins in Arabidopsis, expression of *CYCA3* is S phase specific, as is the single copy in Marchantia and animals, and its overexpression affects meristem activity in Arabidopsis (Takahashi et al. 2010; Willems et al. 2020). This suggests that controlling cell cycle progression during S phase could be the ancestral role for A-type cyclins across plants. Subsequently, roles in ploidy and meiosis could have evolved later associated with the *CYCA2* and *CYCA1* clades, respectively.

B-type cyclins, function, and phase specificity are well conserved across eukaryotes, playing a key role in regulating the G2/M transition (Pecani et al. 2022), as we also observed in Marchantia. In addition, we showed that MpCYCB;1 translocates into the nucleus during metaphase-anaphase, contributing to a spike of cyclin activity in the nuclei during the G2/M transition. The translocation of B-type cyclins, which promotes disassembly of the nuclear envelope breakdown, has been well described in animal cells (Clute and Pines 1999; Gavet and Pines 2010) by the active export of CYCB from the nucleus to the cytosol during the interphase (Toyoshima et al. 1998; Hagting et al. 1999). While some CYCB are found both in the nuclei and cytosol in plants, it is often associated with condensing chromosomes after nuclear envelope disassembly (Criqui et al. 2001; John et al. 2001; Boruc et al. 2010b). CYCB translocation works very differently in fission yeast, where cdc13 is localized in the nuclei until it is exported to the cytosol before mitosis (Kapadia and Nurse 2025). The possibility that this mechanism might be conserved between animals and plants might indicate it is ancestral to most eukaryotes.

Overexpression of *CYCB* has been associated with higher division rates (Doerner et al. 1996). However, in Marchantia, overexpression of Mp*CYCB;1* or Mp*CYCA* did not increase division rates but instead produced arrest. CYCB1;1 induction in Arabidopsis has also been shown to cause mitotic arrest under DNA damage conditions (Schnittger and De Veylder 2018). In animals, B-type cyclins are also not rate limiting for cell proliferation (Doonan and Hunt 1996), suggesting that this could represent the ancestral state of CYCB protein function in eukaryotes. However, further studies are required to generalize the precise functions of A- and B-type cyclins in plants.

The balance between cell division or arrest is highly dose dependent. Cell cycle arrest may result from the inability of some cells to degrade excessive cyclins or additional checkpoint triggering arrest, thereby preventing progression through mitosis (Fig. 5h). The expression of nondegradable CYCBs in plants and other eukaryotes has also been shown to cause mitotic arrest (Chang et al. 2003; Weingartner et al. 2004), supporting the view that their degradation by the APC/C is critical (Fig. 6). Similarly, A-type cyclins also need to be degraded by the APC/C but prior to the spindle assembly checkpoint in other eukaryotes (Geley et al. 2001). Our results suggest that CYCA degradation is critical for cell cycle progression in plants as well (Fig. 5).

On the other hand, our data indicate that MpCYCD;1 is the single rate-limiting cyclin/CDK component for cell proliferation in Marchantia (Fig. 5). Unlike MpCYCA and MpCYCB;1, its degradation may not be required for mitotic progression. Rather, its accumulation triggers re-entry into G1 and may be crucial for integrating developmental and environmental signals to regulate plant growth rates.

The role of the DREAM complex TFs (E2F/DP and 3R-MYBs) is also important to shape the downstream signaling networks of the cell cycle. The Marchantia genome encodes homologs of these regulators, but only 1 copy of each component is expressed in the gametophyte. The single-copy nature of E2F TFs in Marchantia and other land plants suggests that the complexity observed in Arabidopsis and animals arose independently (Sozzani et al. 2006; Rauber et al. 2016). The diversification of 3R-MYBs appears to be ancestral to land plants, and their expansion in Marchantia may be linked to sporophyte-specific programs (Dataset S1).

CKI are also conserved across eukaryotes, with distinct roles in regulating cell cycle progression. In *Marchantia*, we showed that *MpKRP* can inhibit cell division, leading to bigger cells. This role is reminiscent of the mammalian Cip/Kip *p21* blocking of DNA replication by inhibiting CDK activity (Funk et al. 1997) and Arabidopsis KRP (De Veylder et al. 2001b). In Marchantia there is no endoreduplication (Nishihama et al. 2015), suggesting they function independent of it. We found that the lack of MpKRP can generate smaller cells but with little effect in cell differentiation. This is consistent with higher order mutants in *Arabidopsis* (Sizani et al. 2019). A similar phenotype was also observed in Marchantia SIAMESE-related mutants (Hernandez-Garcia et al. 2021). We also found that overexpression of WEE also arrest cell cycle (De Schutter et al. 2007). This supports the idea that WEE and KRP are modulators of plant growth but not required for cell cycle progression or normal development. In general, most components are expected to be functionally conserved. Yet there are several components not analyzed in detail here. Among them, the presence of a potential canonical Mp*CDC25;1* is a good example of components that might work differently in land plants. In conclusion, Marchantia provides a valuable model for studying cell cycle to demonstrate general principles of growth regulation that could be applied to complex organisms. While our overexpression studies provide insights into cyclin function, loss-of-function approaches using conditional knockouts (Nishihama et al. 2016) will be necessary to definitively establish essentiality. Targeted studies examining how environmental stress, DNA damage, hormonal signals, and mechanical cues impact cell cycle progression in Marchantia will yield insights into the evolutionary adaptation of this vital process. The foundational work presented here establishes a toolkit of genes and promoters that can be leveraged not only for fundamental research but also for biotechnological applications. The simplified system described here provides an ideal platform for synthetic biology approaches to engineering plant growth. A deeper understanding of the cell cycle will enable more precise approaches to reprogramming plant growth and development.

## Materials and methods

### Phylogenetic analysis

*Ceratopteris richardii* v2.1 (Marchant et al. 2022), *Amborella trichopoda* v1.0 (Amborella Genome 2013), *Chlamydomonas reinhardtii* v5.5 (Merchant et al. 2007), *Physcomitrium patens* v6.1 (Bi et al. 2024), and *Spirodela polyrhiza v2* (Wang et al. 2014) proteomes were obtained from Phytozome. *Phaeodactylum tricornutum* ASM15095 v2 (Bowler et al. 2008), *Prasinoderma coloniale* v1.1 (Li et al. 2020b), *Ostreococcus lucimarinus* ASM9206 v1 (Palenik et al. 2007), *Mesotaenium endlicherianum* v2 (Cheng et al. 2019), *Mesostigma viridea* (Liang et al. 2020), *Ectocarpus siliculosus* (Cock et al. 2010), and *Chara braunii* v1.0 (Nishiyama et al. 2018) proteomes were obtained from Phycocosm. *Saccharomyces cerevisiae* R64.1.1 (Goffeau et al. 1996), *Caenorhabditis elegans* (Consortium 1998), and *Drosophila melanogaster* BDGP6.46 (Adams et al. 2000) proteomes were obtained from Ensembl, and *Cyanidioschyzon merolae* ASM9120 v1 (Matsuzaki et al. 2004) was obtained from Ensembl plants. The proteomes from *Marchantia polymorpha Tak* v6.1 (Montgomery et al. 2020), *Selaginella kraussiana* v2 (Liu et al. 2023), *Anthoceros agresti* Oxford (Li et al. 2020a), *Klebsormidium nitens* NIES-2285 (Hori et al. 2014), *Zygnema circumcarinatum* (Feng et al. 2024), *Penium margaritaceum* (Jiao et al. 2020), *Marchantia paleacea* (Radhakrishnan et al. 2020), and *Lunularia cruciata* v1 (Linde et al. 2020) were from their respective sources.

Hidden Markov models matrix for PFAMs including cyclins (PF00134.28), CKS (PF01111.24), E2F (PF02319.25), RBR (PF01858.22), KRP (PF02234.24), WEE (PTHR11042), CDC20 (PTHR19918), and CDC25 (PTHR10828) were used as query for searching using hmmsearch function from HMMER v3.4 (Finn et al. 2011). We used the public server at usegalaxy.org to analyze the data (Afgan et al. 2016). The cut-off E-value was then adjusted aiming to have 100% sensitivity for annotated genes and exclude low confidence findings: cyclins < 1E-10, CKS < 1E-10, E2F < 1E-5, RBR < 1E-50, WEE < 1E-45, CDC20 < 1E-50, CDC25 < 1E-20. Using the hmmsearch output, Seqinr in R (Charif and Lobry 2007) package was to retrieve the sequences from the proteomes.

3R-MYBs proteins were BLASTed using the human MYBB sequence as a query (threshold E-value < 2e-34) aiming to have 100% sensitivity for annotated 3R-MYB and include some R2R3-MYBs. Similarly, for CDK proteins, we used yeast CDC28 and Arabidopsis CDKF as query (threshold E-value < 1e-44), aiming to have 100% sensitivity for annotated CDKs. Sequences were extracted from the proteomes using Biostrings package in R (Lifschitz et al. 2022).

Finally, JalView (Procter et al. 2021) was used to align sequences with the MAFFT software (Katoh et al. 2002) and trimmed the alignment to include only columns with 50% coverage. Maximum-likelihood phylogenetic analysis was performed using IQtree 2 (Minh et al. 2020) with default parameters. Trees were rooted and visualized using iTOL v6 (Letunic and Bork 2024). Complete trees available at (Dataset S1).

To count the number of MYB repeats (SM00717) for 3R-MYB proteins, we used InterProScan (Quevillon et al. 2005). To identify signature motifs on CDKs we used a simple grepl search function. Marchantia genes names were annotated in MarpolBase.

### Analysis of RNA-sequencing data

TPM values for sporeling germination and different developmental stages were extracted from Marpolbase Expression database (Kawamura et al. 2022) or downloaded from SRA for regeneration (DRR330148- DRR330173), mapped in *M. polymorpha* Tak-1 genome v5.1 using HISAT2 (Kim et al. 2019), ht-seq, and EdgeR. Data were subsequently analyzed with R to generate plots using customs scripts. Heatmap was generated using Marpolbase Expression database (Kawamura et al. 2022).

The scRNA-seq matrix for Marchantia gemmaling development (Wang et al. 2023) was extracted from Beijing Institute of Genomics Data Center (OMIX004749) and reanalyzed using Seurat V5 package in R (Hao et al. 2024). Briefly, the dataset was subsetted to only cluster 10 and analyzed with the following functions and parameters: FindNeighbours (dims = 1:100, k.param = 200), FindClusters, RunUMAP (reduction = “pca”, metric = “correlation”, n.neighbours = 30L, dims = 1:100). Data were visualized using DimPlot, FeaturePlot, and DotPlot functions. Markers in Table S1 were obtained using FindAllMarkers function in Seurat.

### Bioinformatic analysis

Genes annotated using MarpolBase. For Gene Ontology (GO) analysis, the list of gene names of each subcluster (Table S1) was with the built-in function for GO enrichment in MarpolBase Expression (Kawamura et al. 2022) and gene orthology analysis were done using eggNOG-mapper v2 and eggNOG 5.0 database (Huerta-Cepas et al. 2019; Cantalapiedra et al. 2021).

For protein structure prediction and bimolecular interactions, the full length of the amino acid sequence of either a single or 2 proteins was submitted to the AlphaFold 3 server (Abramson et al. 2024) using default parameters. The first model was used to visualize, and ipTM values were used as a proxy for the confidence of the interaction.

### Plant material and growth conditions

*Marchantia polymorpha* subs*. rudelaris* accessions *Cam-1* (male) and *Cam-2* (female) were used for most of the experiments. Under normal conditions, plants were grown on solid 0.5× Gamborg B-5 basal medium (Phytotech #G398) at pH 5.8 with 1.2% (w/v) agar micropropagation grade (Phytotech #A296), under continuous LED light at 21 °C with light intensity of 150 μmol/m^2^/s (Systion #SE-EGB). For spore production, plants were grown in Microbox micropropagation containers (SacO_2_) in long-day conditions (16 h light/8 h dark) under light supplemented with far-red light as described (Sauret-Gueto et al. 2020).

For Mp*cycd;2* knock-outs, the *M. polymorpha* subs*. rudelaris* male accession Takaragaike-1 (Tak-1) accession was used as wild type. Similarly, *M. polymorpha* plants were grown on half-strength Gamborg B5 medium (pH 5.5) solidified with 1.4% (w/v) agar at 22 °C under continuous light.

### Plasmid construction

To generate new L0 parts, CDS, and promoter regions from genes were extracted from *M. polymorpha* Tak-1 genome version 5.1 (Bowman et al. 2017) genome and manually domesticated to remove internal BsaI and SapI sites using synonymous mutations for the CDS. The sequences of synthetic L0 parts used in this work are available in Table S2. L0 parts were synthesized by GENEWIZ following the standard syntax for plant synthetic biology with CDS and PROM5 or PROM and 5UTR overhangs and cloned into the plasmid pUAP1 (Addgene #63674) (Patron et al. 2015) by homology recombination. Other parts were derived from previous works (Sauret-Gueto et al. 2020; Romani et al. 2024) as specified in Table S2.

The acceptor pBy01 (Romani et al. 2024) was used to assemble using L0 corresponding to PROM5 or PROM and 5UTR parts and pBy10 (Tse et al. 2024) for entire cassettes containing a hygromycin-resistant cassette for plant selection. These acceptors are binary vectors that contain a LacZ selection cassette flanked by BsaI sites to clone final vectors in 1 step using L0 parts following the standard syntax (Patron et al. 2015). Following the same logic, a new custom acceptor for chlorsulfuron selection was generated based on pBy10 and OP-62 (Sauret-Gueto et al. 2020; Romani et al. 2024). Another 2 custom acceptors were generated for cloning only CDS with the *_pro_35S* (*pBy12*) or estradiol inducible system using *_pro_MpE2F:XVE* driving the expression of *_pro_LexA,* similar to previous published Gateway acceptor pMpGWB168 (Ishida et al. 2022) and a *_3utr_NOS* terminator, containing also hygromycin (*pBy13*) or chlorsulfuron (*pBy23*) resistant cassette for plant selection. The full length of the final constructs were verified by sequencing using the Oxford Nanopore technology (Plasmidsaurus Inc.). The final plasmid map is provided in the supplementary data (Datasets S2 and 3).

Plasmids were assembled as detailed in Table S2 using Type-IIS cloning as described previously for L3 plasmids (Romani et al. 2024). Briefly using a Master Mix containing 10% (v/v) 10× T4 DNA ligase buffer (NEB #M0202), 5% (v/v) T4 DNA ligase at 400 U/μL (NEB #M0202), 5% (v/v) BsaI at 20 U/μL (NEB #R3733), 10% (v/v) acceptor at 40 ng/μL, and 20% (v/v) premixed L0 parts (∼100 ng/μL) and water to a final volume of 5 μL. Cycling conditions were 26 cycles of 37 °C for 3 min and 16 °C for 4 min. Termination and enzyme denaturation: 50 °C for 5 min, and 80 °C for 10 min. Other L1 and L2 plasmids were cloned as described before (Sauret-Gueto et al. 2020). TOP10 chemically competent *Escherichia coli* cells were transformed using the assembly reaction and L0 parts as described in detail in Table S2 for each construct. The presence of the correct insert was confirmed by restriction XhoI digestion (Thermo Scientific #FD0694) and Sanger sequencing, with primers available at Table S2.

For genome editing of Mp*CYCD;2* (*Mp1g24670*), a guide RNA was designed to target the coding sequence of the first exon using CRISPRdirect (Naito et al. 2015). For MpKRP a guide RNA targeting the active domain of the protein. The plasmids were constructed according to Sugano et al., using pMpGE_En03 and pMpGE010 (Sugano et al. 2018). Primers for gRNA and genotyping are shown at Table S2.

### Plant transformation

*Agrobacterium tumefaciens* (GV3101) cells were transformed using the freeze-thaw method and used for plant transformation of *Cam-1/2* spores as described in Annese et al. (2025) and selected for either 20 μg/mL hygromycin (Invitrogen #10687010) or 0.5 μM chlorsulfuron (Thermo Scientific #17959385). Plants were screened for positive fluorescence and at least 2 independent lines were selected. For expression markers, representative transgenic lines are shown, showing the consensus expression pattern out of 4 to 5 independent lines. In the case of bimolecular fluorescence complementation (BiFC), plants were co-transformed with 2 *A. tumefaciens* strains harboring each plasmid and selected for hygromycin and chlorsulfuron to obtain stable transgenic lines. At least a dozen independent transformant sporelings were analyzed for fluorescent protein reconstruction to obtain consistent results for protein-protein interactions.

For genome editing of Mp*CYCD;2*, Tak-1 plants were transformed using the cutting thallus transformation protocol (Kubota et al. 2013) and genotyped using primers shown at Table S2.

### Plant phenotyping

Representative images of Marchantia plants were taken using a Keyence VHX-5000 digital microscope equipped with a 20×-200× Ultra-Small, High-Performance Zoom Lens (VH-Z20R/Z20T) or a Leica DMS1000 digital microscope. Thallus area was quantified from pictures (normally 7-d-old gemmalings) with ImageJ software with at least 10 biological replicates. Cell sizes were quantified from manual segmentation of cells in 200× pictures using ImageJ. Only cells surrounding the DDCZ but excluding smaller cells closer to the apical meristem. At least 50 cells were measured for each biological replicate in at least 3 biological replicates.

For β-estradiol (Merck #E8875) treatments, a 50-mM stock was dissolved in DMSO and added to melted agar-Gamborg B-5 media at a final concentration of 5 mM as described in Ishida et al. (2022). Plants were grown in microscopy contact plates and visualized after 3 or 10 d in normal growth conditions.

### Laser scanning confocal microscopy

Confocal images of Marchantia were acquired on a Leica SP8X spectral confocal microscope upright system equipped with a 460- to 670-nm super continuum white light laser (80% laser power), 2 CW laser lines 405, and 442 nm, and 5 Channel Spectral Scanhead (four hybrid detectors and 1 photomultiplier). For slides, imaging was conducted using either a 10× air objective (HC PL APO 10×/0.40 CS2) or a 20× air objective (HC PL APO 20×/0.75 CS2). When observing fluorescent protein with overlapping emission spectra, sequential scanning mode was selected. Excitation laser wavelength and captured emitted fluorescence wavelength window as following: mVenus (514 nm, 527 to 552 nm), and for chlorophyll autofluorescence (633, 687 to 739 nm).

### Time-course and time-lapses

When imaging time-courses, plants were grown under normal culture conditions in contact plates (Simport Scientific #SIMPD210-17), the lid was removed for imaging, and plants were returned to the growth chamber and imaged as described above. For live imaging, 6 stacked Gene Frames (ThermoFisher #AB0578,) were placed on a glass slide and filled halfway with molten Gamborg B-5 agar medium. Plants were then placed on the solidified agar surface, and meristems were removed using a Laser Microdissection Leica LMD7000. Samples were mounted in perfluorodecalin (Sigma-Aldrich #P9900) (Littlejohn et al. 2010) with a glass coverslip on top. The slides were then continuously imaged on the Leica SP8X confocal microscope for 1 to 4 d.

### EdU labeling

The 5-d-old gemmalings were incubated in liquid half-strength Gamborg B-5 medium under continuous light for 3 h with 20 μM 5-ethynyl- 2′-deoxyuridine (EdU) from the Click-iT EdU Imaging kit with Alexa Fluor 488 (Invitrogen #C10337). Then they were fixed with 4% formaldehyde for 1 h and washed twice with phosphate buffer saline (PBS) and 0.5% Triton X-100 in PBS for 20 min. After washes, samples were incubated with a freshly prepared reaction mixture following the manufacturer instructions but without the Hoechst 33,342 component. After labeling, samples were protected from light, washed twice with PBS and soaked in iTomei-D (Tokyo Chemical Industry #T3940) clearing solution and mounted in 70% w/v iohexol (Tokyo Chemical Industry #I0903) in PBS as described before (Sakamoto et al. 2022). Samples were covered with a glass coverslip and imaged on the Leica SP8X confocal microscope as described before.

### Statistics

To obtain plots and statistical analysis, data was processed using R version 4.4.1 software. For average and boxplots, the stats package was used with default parameters. In each boxplot, the central line indicating the median, box limits represent the first and third quartiles (interquartile range, IQR). Individual datapoints are indicated. For violin plots, the vioplot package was implemented also with default parameters. Statistical significance was calculated using ANOVA using the stats package Tukey honest significant difference method (alpha = 0.05) for levels calculations.

### Accession numbers

Naming is consistent nomenclature guidelines (Bowman et al. 2016). Information about genes was submitted to MarpolBase (https://marchantia.info) and it is available in Table S2.

## Supplementary Material

koag103_Supplementary_Data

## Data Availability

The data underlying this article will be shared on reasonable request to the corresponding author. RNA-seq data underlying this article are available in MarpolBase Expression, at https://mbex.marchantia.info. The scRNA-seq datasets were derived from sources in the public domain: PRJCA013186.
