# Supplementary material for: A simple cell-cycle control system in Marchantia polymorpha provides a framework for understanding plant cell proliferation
Source: Plant Cell. 2026 Apr 9;38(6):koag103. doi: 10.1093/plcell/koag103 (PMC13237563; doi:10.1093/plcell/koag103)
Supplement: koag103_Supplementary_Data [file koag103_supplementary_data.zip › Supplementary Dataset S1/Phylogenetic trees.pdf]

***Supplemental File 1.*** *Supporting information for phylogenetic analysis of cell-cycle proteins. (a) Phylogeny of Cyclins. (b) Phylogeny of the CDK and PSTAIRE motifs. (c) Phylogeny of CKS proteins. (d) Phylogeny of E2F/DP/DEL proteins. (e) Phylogeny of RBR. (f) Phylogeny of 3R-MYBs and analysis of sequence repeats. (g) Phylogeny of CDC20/CDH1 proteins. (h) Phylogeny of CDC25 proteins. (i) Phylogeny of WEE proteins. (j) Phylogeny of KRP proteins.*

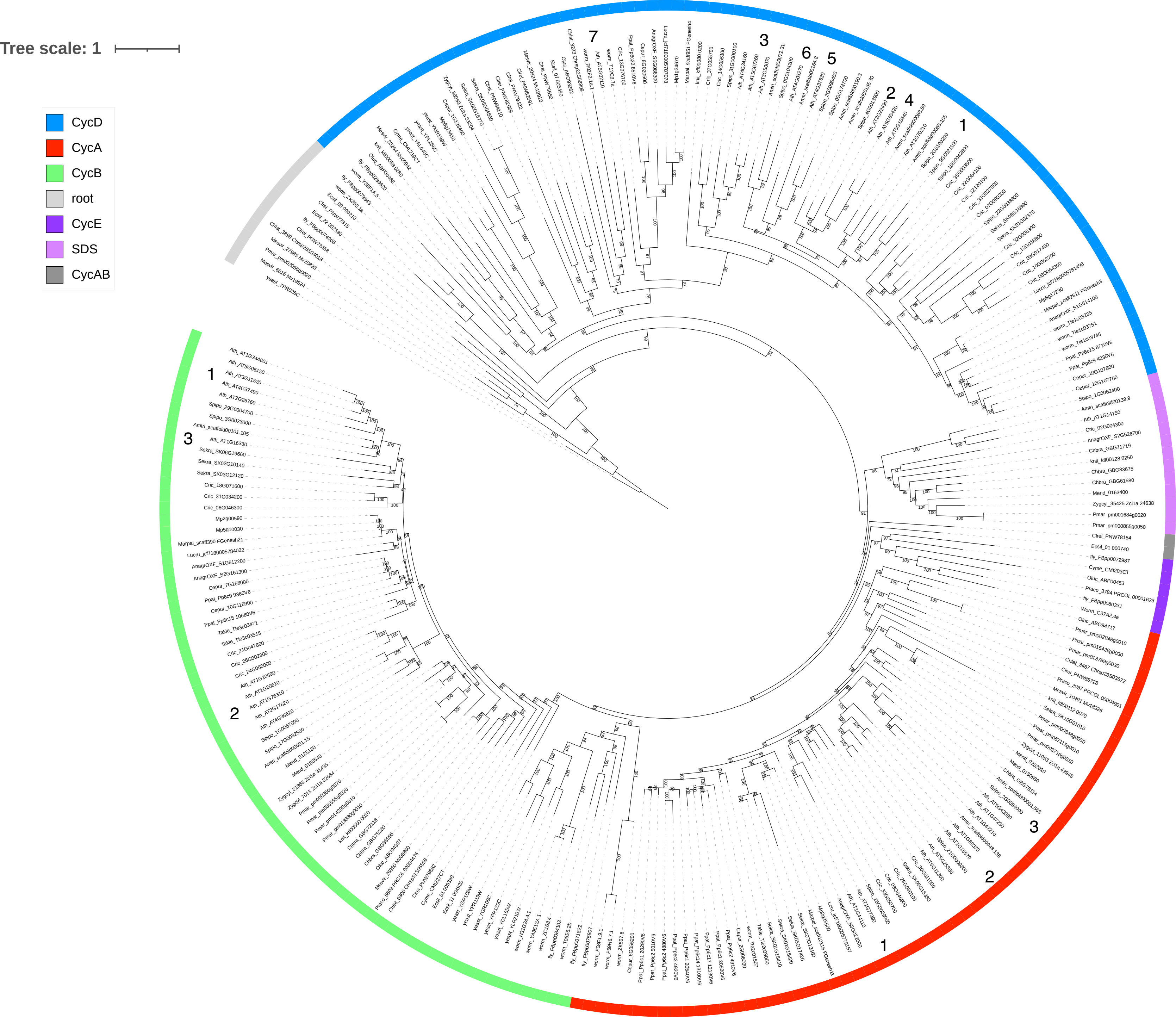

Tree scale: 1
CycD
CycA
CycB
root
CycE
SDS
CycAB

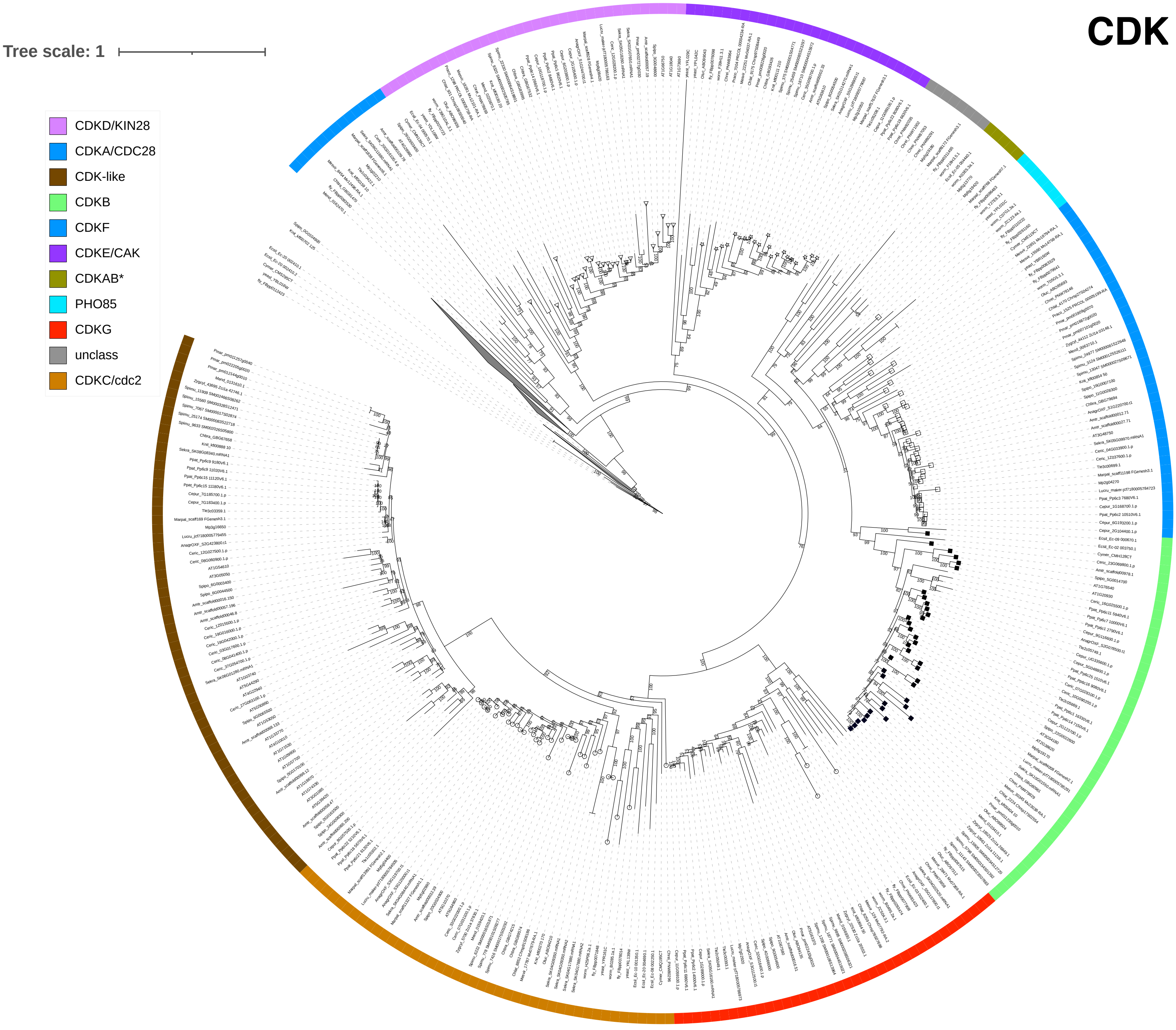

CDK
Tree scale: 1
CDKD/KIN28
CDKA/CDC28
CDK-like
CDKB
CDKF
CDKE/CAK
CDKAB*
PHO85
CDKG
unclass
CDKC/cdc2

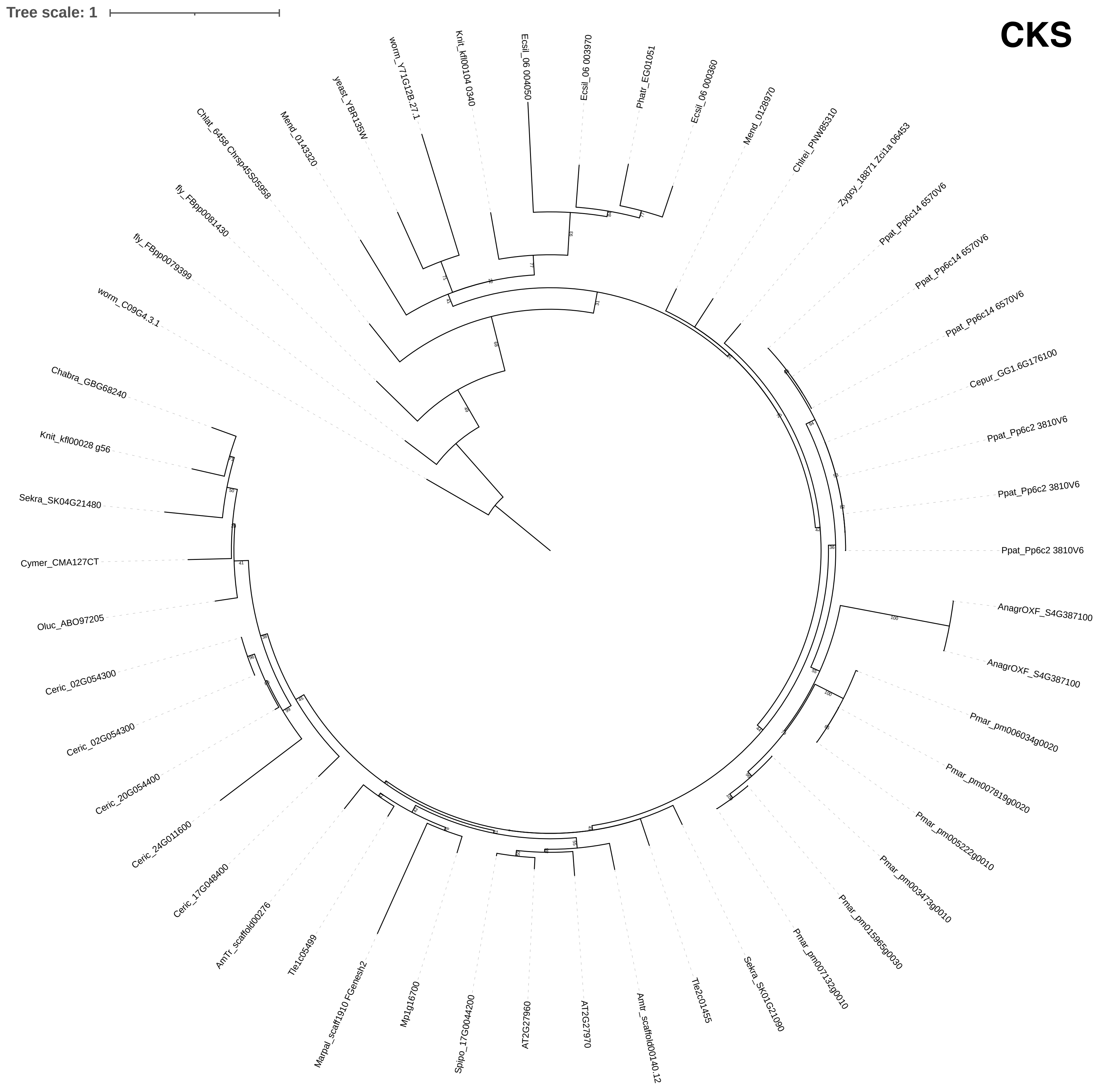

Tree scale: 1
CKS
Ecsil_06 004050
Ecsil_06 003970
Phatr_EG01051
Ecsil_06 000360
Mend_0128970
Chlrei_PNW85310
Zygcy_18871 Zci1a 06453
Ppat_Pp6c14 6570V6
Ppat_Pp6c14 6570V6
Ppat_Pp6c14 6570V6
Cepur_GG1.6G176100
Ppat_Pp6c2 3810V6
Ppat_Pp6c2 3810V6
Ppat_Pp6c2 3810V6
AnagrOXF_S4G387100
AnagrOXF_S4G387100
Pmar_pm006034g0020
Pmar_pm007819g0020
Pmar_pm005222g0010
Pmar_pm003473g0010
Pmar_pm015965g0030
Pmar_pm007132g0010
Sekra_SK01G21090
Tle2c01455
Amtr_scaffold00140.12
AT2G27970
AT2G27960
Spipo_17G0044200
Mp1g16700
Marpal_scaff1910 FGenesh2
Tle1c05499
AmTr_scaffold00276
Ceric_17G048400
Ceric_24G011600
Ceric_20G054400
Ceric_02G054300
Ceric_02G054300
Oluc_ABO97205
Cymer_CMA127CT
Sekra_SK04G21480
Knit_kfl00028 g56
Chabra_GBG68240
worm_C09G4.3.1
fly_FBpp0079399
fly_FBpp0081430
Chlat_6458 Chrsp45S05958
Mend_0143320
yeast_YBR135W
worm_Y71G12B.27.1
Knit_kfl00104 0340

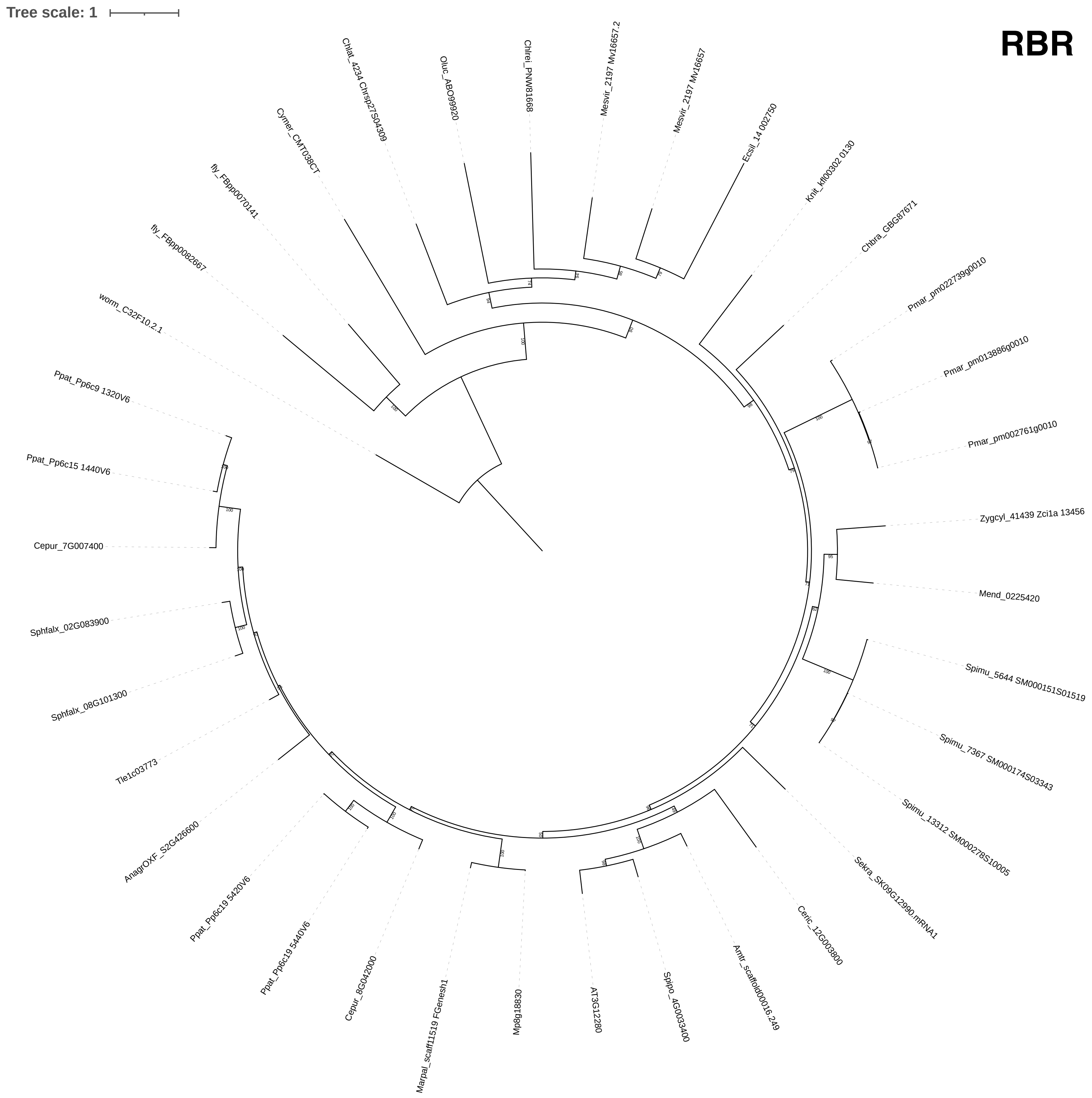
Tree scale: 1
RBR
Chlat_4234 Chrsp27S04309
Oluc_ABO99920
Chlrei_PNW81668
Mesvir_2197 Mv16657.2
Mesvir_2197 Mv16657
Ecsil_14 002750
Knit_kfl00302 0130
Chbra_GBG87671
Pmar_pm022739g0010
Pmar_pm013886g0010
Pmar_pm002761g0010
Zygcyl_41439 Zci1a 13456
Mend_0225420
Spimu_5644 SM000151S01519
Spimu_7367 SM000174S03343
Spimu_13312 SM000278S10005
Sekra_SK09G12990.mRNA1
Ceric_12G003800
Amtr_scaffold00016.249
Spipo_4G0033400
AT3G12280
Mp8g18830
Marpal_scaff11519 FGenesh1
Cepur_8G042000
Ppat_Pp6c19 5440V6
Ppat_Pp6c19 5420V6
AnagrOXF_S2G426600
Tle1c03773
Sphfalx_08G101300
Sphfalx_02G083900
Cepur_7G007400
Ppat_Pp6c15 1440V6
Ppat_Pp6c9 1320V6
worm_C32F10.2.1
fly_FBpp0082667
fly_FBpp0070141
Cymer_CMT038CT

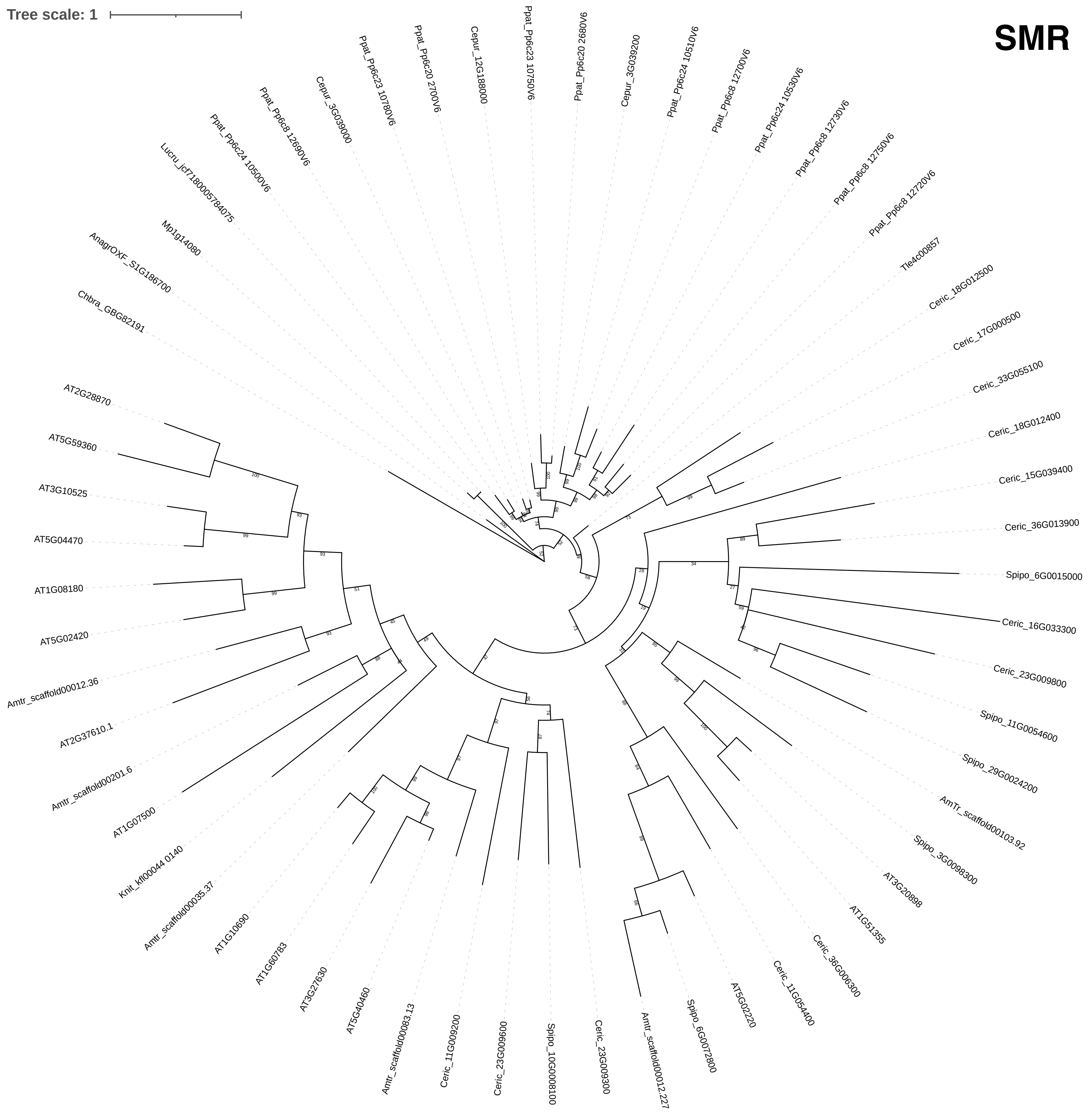
Tree scale: 1
SMR
Chbra_GBG82191
AnagrOXF_S1G186700
Mp1g14080
Lucru_jcf7180005784075
Ppat_Pp6c24 10500V6
Ppat_Pp6c8 12690V6
Cepur_3G039000
Ppat_Pp6c23 10780V6
Ppat_Pp6c20 2700V6
Cepur_12G188000
Ppat_Pp6c23 10750V6
Ppat_Pp6c20 2680V6
Cepur_3G039200
Ppat_Pp6c24 10510V6
Ppat_Pp6c8 12700V6
Ppat_Pp6c24 10530V6
Ppat_Pp6c8 12730V6
Ppat_Pp6c8 12750V6
Ppat_Pp6c8 12720V6
Tle4c00857
Ceric_18G012500
Ceric_17G000500
Ceric_33G055100
Ceric_18G012400
Ceric_15G039400
Ceric_36G013900
Spipo_6G0015000
Ceric_16G033300
Ceric_23G009800
Spipo_11G0054600
Spipo_29G0024200
AmTr_scaffold00103.92
Spipo_3G0098300
AT3G20898
AT1G51355
Ceric_36G006300
Ceric_11G054400
AT5G02220
Spipo_6G0072800
Amtr_scaffold00012.227
Ceric_23G009300
Spipo_10G0008100
Ceric_23G009600
Ceric_11G009200
Amtr_scaffold00083.13
AT5G40460
AT3G27630
AT1G60783
AT1G10690
Amtr_scaffold00035.37
Knit_kfl00044 0140
AT1G07500
Amtr_scaffold00201.6
AT2G37610.1
Amtr_scaffold00012.36
AT5G02420
AT1G08180
AT5G04470
AT3G10525
AT5G59360
AT2G28870

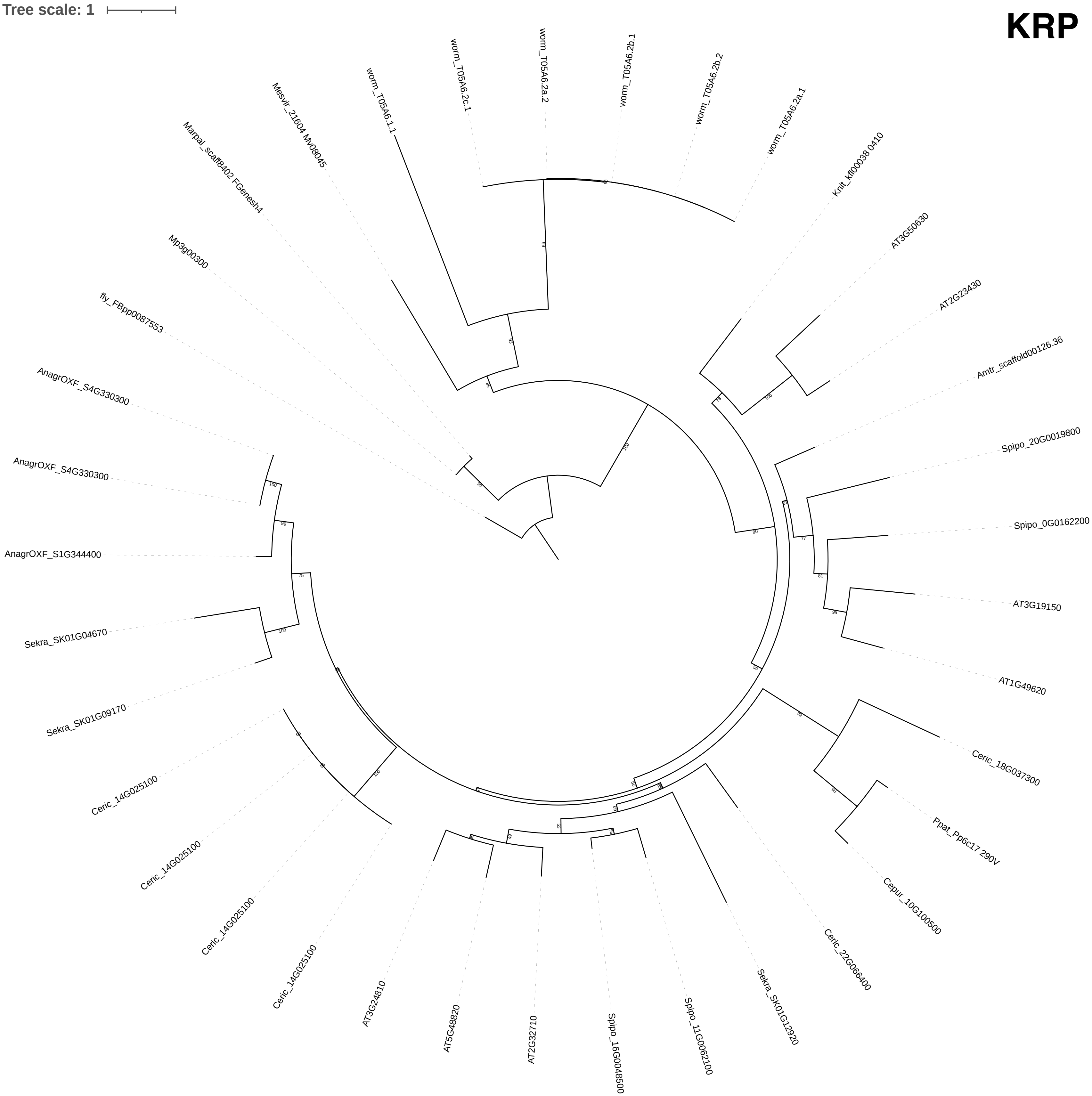
Tree scale: 1
KRP
worm_T05A6.1.1
worm_T05A6.2c.1
worm_T05A6.2a.2
worm_T05A6.2b.1
worm_T05A6.2b.2
worm_T05A6.2a.1
Knit_kfl00038 0410
AT3G50630
AT2G23430
Amtr_scaffold00126.36
Spipo_20G0019800
Spipo_0G0162200
AT3G19150
AT1G49620
Ceric_18G037300
Ppat_Pp6c17 290V
Cepur_10G100500
Ceric_22G066400
Sekra_SK01G12920
Spipo_11G0062100
Spipo_16G0048500
AT2G32710
AT5G48820
AT3G24810
Ceric_14G025100
Ceric_14G025100
Ceric_14G025100
Ceric_14G025100
Sekra_SK01G09170
Sekra_SK01G04670
AnagrOXF_S1G344400
AnagrOXF_S4G330300
AnagrOXF_S4G330300
fly_FBpp0087553
Mp3g00300
Marpal_scaff8402 FGenesh4
Mesvir_21604 Mv08045

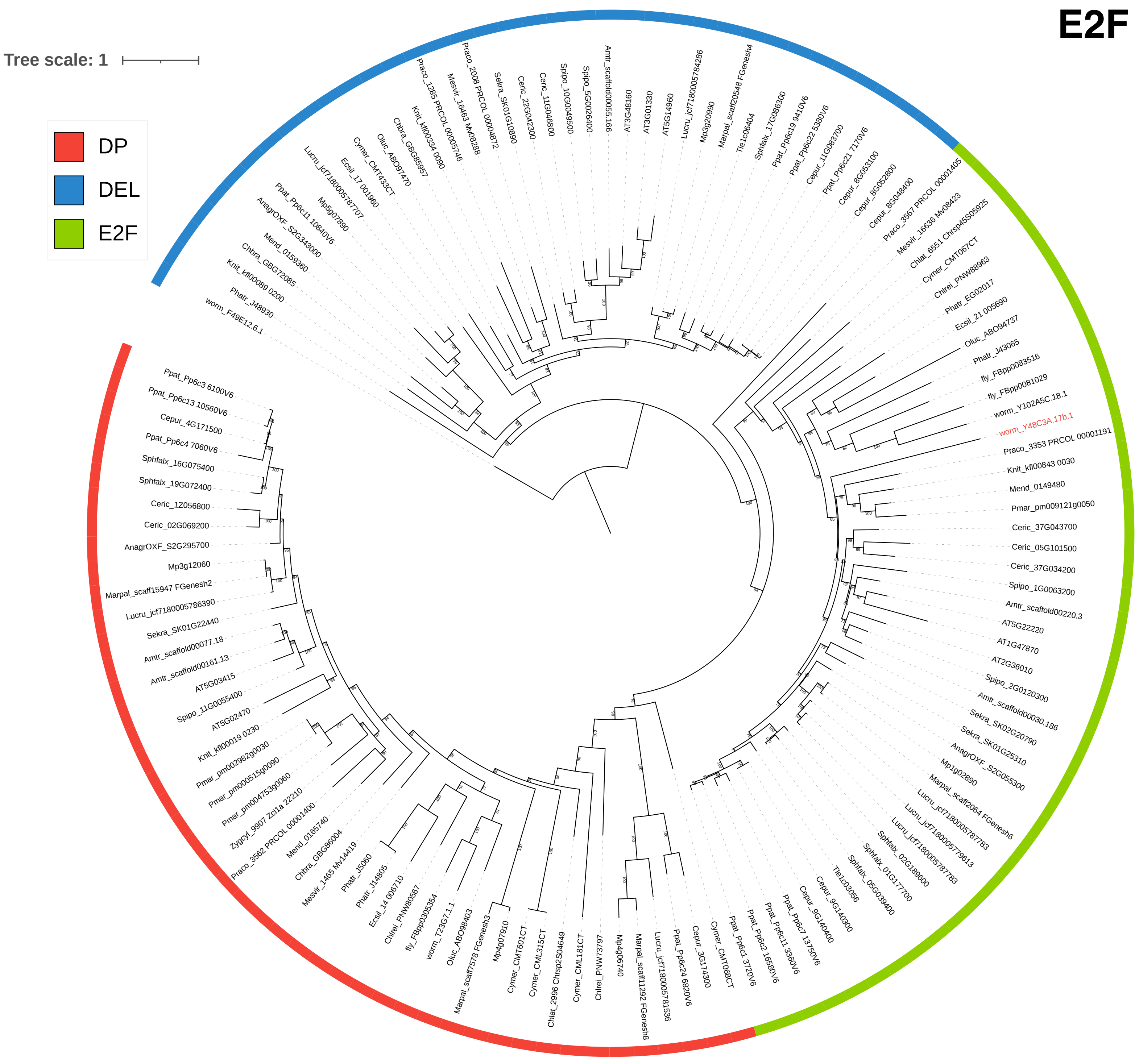
E2F
Tree scale: 1
DP
DEL
E2F
worm_F49E12.6.1
Phatr_J48930
Knit_kfl00089 0200
Chbra_GBG72085
Mend_0159360
AnagrOXF_S2G343000
Ppat_Pp6c11 10840V6
Mp5g07890
Lucru_jcf7180005787707
Ecsil_17 001960
Cymer_CMT433CT
Oluc_ABO97470
Chbra_GBG85957
Knit_kfl00334 0090
Praco_1285 PRCOL 00005746
Mesvir_16463 Mv08288
Praco_2008 PRCOL 00004872
Sekra_SK01G10890
Ceric_22G042300
Ceric_11G046800
Spipo_10G0049500
Spipo_5G0026400
Amtr_scaffold00055.166
AT3G48160
AT3G01330
AT5G14960
Lucru_jcf7180005784286
Mp3g20990
Marpal_scaff20548 FGenesh4
Tle1c06404
Sphfalx_17G086300
Ppat_Pp6c19 9410V6
Ppat_Pp6c22 5380V6
Cepur_11G083700
Ppat_Pp6c21 7170V6
Cepur_8G053100
Cepur_8G052800
Cepur_8G048400
Praco_3567 PRCOL 00001405
Mesvir_16636 Mv08423
Chlat_6551 Chrsp45S05925
Cymer_CMT067CT
Chlrei_PNW88963
Phatr_EG02017
Ecsil_21 005690
Oluc_ABO94737
Phatr_J43065
fly_FBpp0083516
fly_FBpp0081029
worm_Y102A5C.18.1
worm_Y48C3A.17b.1
Praco_3353 PRCOL 00001191
Knit_kfl00843 0030
Mend_0149480
Pmar_pm009121g0050
Ceric_37G043700
Ceric_05G101500
Ceric_37G034200
Spipo_1G0063200
Amtr_scaffold00220.3
AT5G22220
AT1G47870
AT2G36010
Spipo_2G0120300
Amtr_scaffold00030.186
Sekra_SK02G20790
Sekra_SK01G25310
AnagrOXF_S2G055300
Mp1g02890
Marpal_scaff2064 FGenesh6
Lucru_jcf7180005787783
Lucru_jcf7180005779613
Lucru_jcf7180005787783
Sphfalx_02G189600
Sphfalx_01G177700
Sphfalx_05G039400
Tle1c03056
Cepur_9G140300
Cepur_9G140400
Ppat_Pp6c7 13750V6
Ppat_Pp6c11 3360V6
Ppat_Pp6c2 16580V6
Ppat_Pp6c1 3720V6
Cymer_CMT068CT
Cepur_3G174300
Ppat_Pp6c24 6820V6
Lucru_jcf7180005781536
Marpal_scaff11292 FGenesh8
Mp4g06740
Chlrei_PNW73797
Cymer_CML181CT
Chlat_2996 Chrsp2S04649
Cymer_CML315CT
Cymer_CMT601CT
Mp4g07910
Marpal_scaff7578 FGenesh3
Oluc_ABO98403
worm_T23G7.1.1
fly_FBpp0305354
Chlrei_PNW80567
Ecsil_14 006710
Phatr_J14805
Phatr_J5060
Mesvir_1465 Mv14419
Chbra_GBG86004
Mend_0165740
Praco_3562 PRCOL 00001400
Zygcyl_9907 Zci1a 22210
Pmar_pm004753g0060
Pmar_pm000515g0090
Pmar_pm002982g0030
Knit_kfl00019 0230
AT5G02470
Spipo_11G0055400
AT5G03415
Amtr_scaffold00161.13
Amtr_scaffold00077.18
Sekra_SK01G22440
Lucru_jcf7180005786390
Marpal_scaff15947 FGenesh2
Mp3g12060
AnagrOXF_S2G295700
Ceric_02G069200
Ceric_1Z056800
Sphfalx_19G072400
Sphfalx_16G075400
Ppat_Pp6c4 7060V6
Cepur_4G171500
Ppat_Pp6c13 10560V6
Ppat_Pp6c3 6100V6

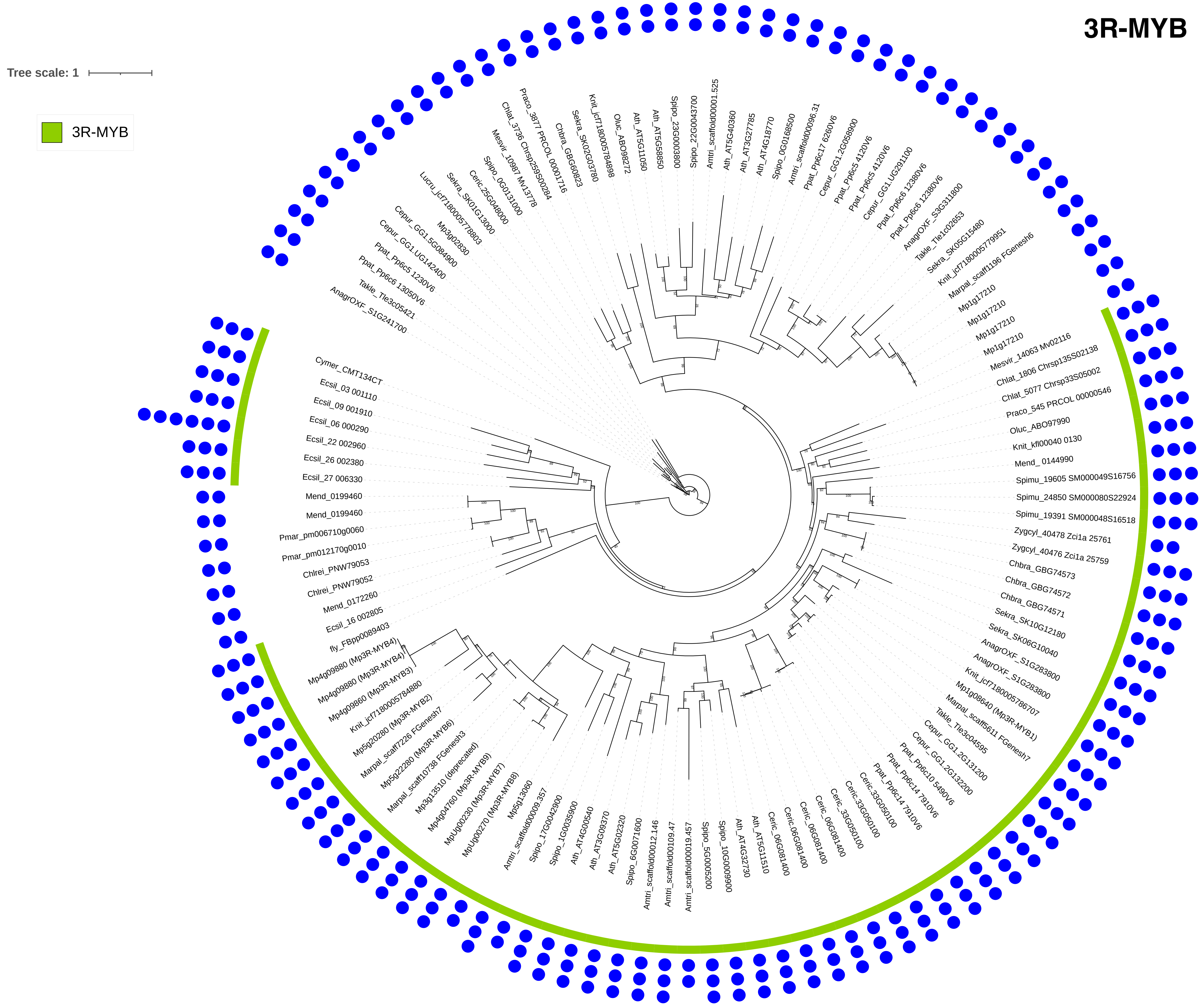

3R-MYB
Tree scale: 1
3R-MYB
Cymer_CMT134CT
Ecsil_03 001110
Ecsil_09 001910
Ecsil_06 000290
Ecsil_22 002960
Ecsil_26 002380
Ecsil_27 006330
Mend_0199460
Mend_0199460
Pmar_pm006710g0060
Pmar_pm012170g0010
Chlrei_PNW79053
Chlrei_PNW79052
Mend_0172260
Ecsil_16 002805
fly_FBpp0089403
Mp4g09880 (Mp3R-MYB4)
Mp4g09880 (Mp3R-MYB4)
Mp4g09860 (Mp3R-MYB3)
Knit_jcf7180005784880
Mp5g20280 (Mp3R-MYB2)
Marpal_scaff7226 FGenesh7
Mp5g22280 (Mp3R-MYB6)
Marpal_scaff10738 FGenesh3
Mp3g13510 (deprecated)
Mp4g04760 (Mp3R-MYB7)
MpUg00230 (Mp3R-MYB9)
MpUg00270 (Mp3R-MYB8)
Mp5g13060
Amtri_scaffold00009.357
Spipo_17G0042900
Spipo_1G0035900
Ath_AT4G00540
Ath_AT3G09370
Ath_AT5G02320
Spipo_6G0071600
Amtri_scaffold00012.146
Amtri_scaffold00109.47
Amtri_scaffold00019.457
Spipo_5G0005200
Spipo_10G0009900
Ath_AT4G32730
Ath_AT5G11510
Ceric.06G081400
Ceric.06G081400
Ceric.06G081400
Ceric.06G081400
Ceric.33G050100
Ceric.33G050100
Ppat_Pp6c14 7910V6
Ppat_Pp6c14 7910V6
Ppat_Pp6c10 5490V6
Cepur_GG1.2G132200
Cepur_GG1.2G131200
Takle_Tle3c04595
Marpal_scaff5611 FGenesh7
Mp1g08640 (Mp3R-MYB1)
Knit_jcf7180005786707
AnagrOXF_S1G283800
AnagrOXF_S1G283800
Sekra_SK06G10040
Sekra_SK10G12180
Chbra_GBG74571
Chbra_GBG74572
Chbra_GBG74573
Zygcyl_40476 Zci1a 25759
Zygcyl_40478 Zci1a 25761
Spimu_19391 SM000048S16518
Spimu_24850 SM000080S22924
Spimu_19605 SM000049S16756
Mend_0144990
Knit_kfl00040 0130
Oluc_ABO97990
Praco_545 PRCOL 00000546
Chlat_5077 Chrsp33S05002
Chlat_1806 Chrsp135S02138
Mesvir_14063 Mv02116
Mp1g17210
Mp1g17210
Mp1g17210
Mp1g17210
Marpal_scaff1196 FGenesh6
Knit_jcf7180005779951
Sekra_SK05G15480
Takle_Tle1c02653
AnagrOXF_S3G311800
Ppat_Pp6c6 12380V6
Ppat_Pp6c6 12380V6
Cepur_GG1.UG291100
Ppat_Pp6c5 4120V6
Ppat_Pp6c5 4120V6
Cepur_GG1.2G058900
Ppat_Pp6c17 6260V6
Amtri_scaffold00096.31
Spipo_0G0168500
Ath_AT4G18770
Ath_AT3G27785
Ath_AT5G40360
Amtri_scaffold00001.525
Spipo_22G0043700
Spipo_23G0003800
Ath_AT5G58850
Ath_AT5G11050
Oluc_ABO98272
Knit_jcf7180005784898
Sekra_SK02G03780
Chbra_GBG60823
Praco_3877 PRCOL 00001716
Chlat_3736 Chrsp259S00284
Mesvir_10987 MV13778
Spipo_0G0131000
Ceric.25G048000
Sekra_SK01G13000
Lucru_jcf7180005778803
Mp3g02830
Cepur_GG1.5G084900
Cepur_GG1.UG142400
Ppat_Pp6c5 1230V6
Ppat_Pp6c6 13050V6
Takle_Tle3c05421
AnagrOXF_S1G241700

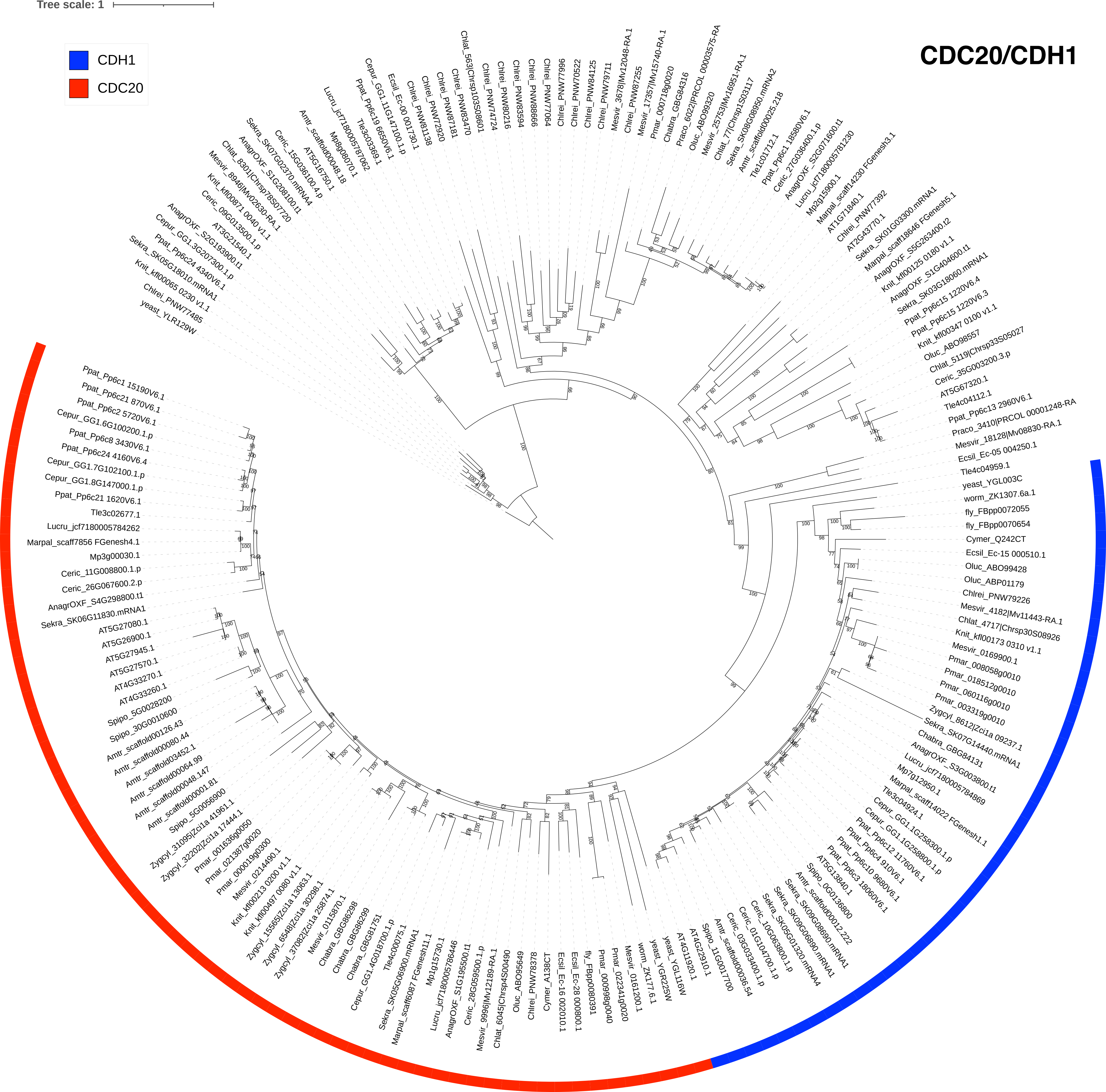

Tree scale: 1
CDH1
CDC20
CDC20/CDH1

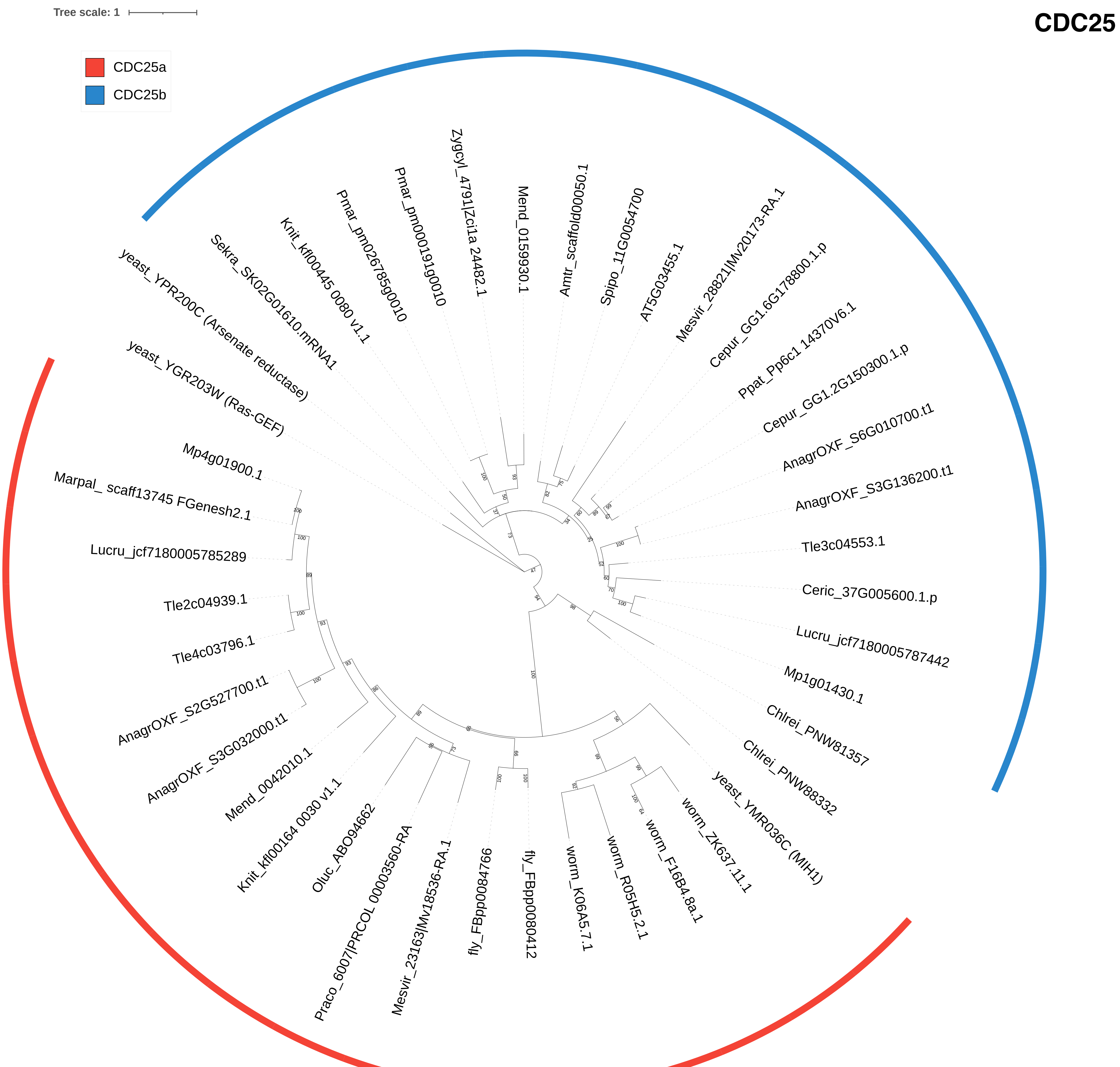

CDC25
Tree scale: 1
CDC25a
CDC25b
Mend_01599930.1
Amtr_scaffold00050.1
Spipo_11G0054700
AT5G03455.1
Mesvir_28821|Mv20173-RA.1
Cepur_GG1.6G178800.1.p
Ppat_Pp6c1 14370V6.1
Cepur_GG1.2G150300.1.p
AnagrOXF_S6G010700.t1
AnagrOXF_S3G136200.t1
Tle3c04553.1
Ceric_37G005600.1.p
Lucru_jcf7180005787442
Mp1g01430.1
Chlrei_PNW81357
Chlrei_PNW88332
yeast_YMR036C (MIH1)
worm_ZK637.11.1
worm_F16B4.8a.1
worm_R05H5.2.1
worm_K06A5.7.1
fly_FBpp0080412
fly_FBpp0084766
Mesvir_23163|Mv18536-RA.1
Praco_6007|PRCOL 00003560-RA
Oluc_ABO94662
Knit_kfl00164 0030 v1.1
Mend_0042010.1
AnagrOXF_S3G032000.t1
AnagrOXF_S2G527700.t1
Tle4c03796.1
Tle2c04939.1
Lucru_jcf7180005785289
Marpal_ scaff13745 FGenesh2.1
Mp4g01900.1
yeast_YGR203W (Ras-GEF)
yeast_YPR200C (Arsenate reductase)
Sekra_SK02G01610.mRNA1
Knit_kfl00445 0080 v1.1
Pmar_pm026785g0010
Pmar_pm000191g0010
Zygcyl_4791|Zci1a 24482.1

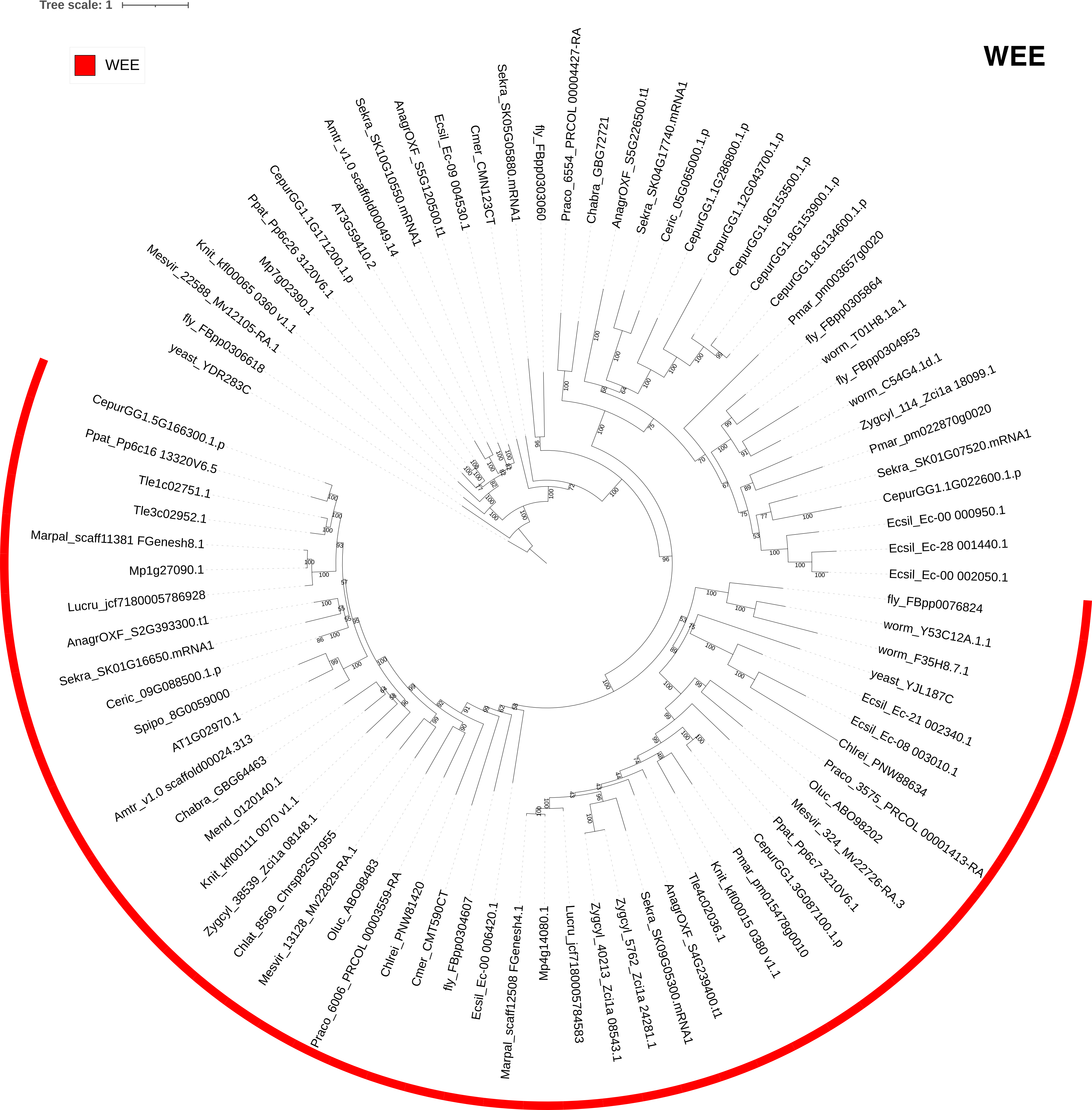

Tree scale: 1
WEE
WEE
yeast_YDR283C
fly_FBpp0306618
Mesvir_22588_Mv12105-RA.1
Knit_kfl00065 0360 v1.1
Mp7g02390.1
Ppat_Pp6c26 3120V6.1
CepurGG1.1G171200.1.p
AT3G59410.2
Amtr_v1.0 scaffold00049.14
Sekra_SK10G10550.mRNA1
AnagrOXF_S5G120500.t1
Ecsil_Ec-09 004530.1
Cmer_CMN123CT
Sekra_SK05G05880.mRNA1
fly_FBpp0303060
Praco_6554_PRCOL 00004427-RA
Chabra_GBG72721
AnagrOXF_S5G226500.t1
Sekra_SK04G17740.mRNA1
Ceric_05G065000.1.p
CepurGG1.1G286800.1.p
CepurGG1.12G043700.1.p
CepurGG1.8G153500.1.p
CepurGG1.8G153900.1.p
CepurGG1.8G134600.1.p
Pmar_pm003657g0020
fly_FBpp0305864
worm_T01H8.1a.1
fly_FBpp0304953
worm_C54G4.1d.1
Zygcyl_114_Zci1a 18099.1
Pmar_pm022870g0020
Sekra_SK01G07520.mRNA1
CepurGG1.1G022600.1.p
Ecsil_Ec-00 000950.1
Ecsil_Ec-28 001440.1
Ecsil_Ec-00 002050.1
fly_FBpp0076824
worm_Y53C12A.1.1
worm_F35H8.7.1
yeast_YJL187C
Ecsil_Ec-21 002340.1
Ecsil_Ec-08 003010.1
Chlrei_PNW88634
Praco_3575_PRCOL 00001413-RA
Oluc_ABO98202
Mesvir_324_Mv22726-RA.3
Ppat_Pp6c7 3210V6.1
CepurGG1.3G087100.1.p
Pmar_pm015478g0010
Knit_kfl00015 0380 v1.1
Tle4c02036.1
AnagrOXF_S4G239400.t1
Sekra_SK09G05300.mRNA1
Zygcyl_5762_Zci1a 24281.1
Zygcyl_40213_Zci1a 08543.1
Lucru_jcf7180005784583
Mp4g14080.1
Marpal_scaff12508 FGenesh4.1
Ecsil_Ec-00 006420.1
fly_FBpp0304607
Cmer_CMT590CT
Chlrei_PNW81420
Praco_6006_PRCOL 00003559-RA
Oluc_ABO98483
Mesvir_13128_Mv22829-RA.1
Chlat_8569_Chrsp82S07955
Zygcyl_38539_Zci1a 08148.1
Knit_kfl00111 0070 v1.1
Mend_0120140.1
Chabra_GBG64463
Amtr_v1.0 scaffold00024.313
AT1G02970.1
Spipo_8G0059000
Ceric_09G088500.1.p
Sekra_SK01G16650.mRNA1
AnagrOXF_S2G393300.t1
Lucru_jcf7180005786928
Mp1g27090.1
Marpal_scaff11381 FGenesh8.1
Tle3c02952.1
Tle1c02751.1
Ppat_Pp6c16 13320V6.5
CepurGG1.5G166300.1.p

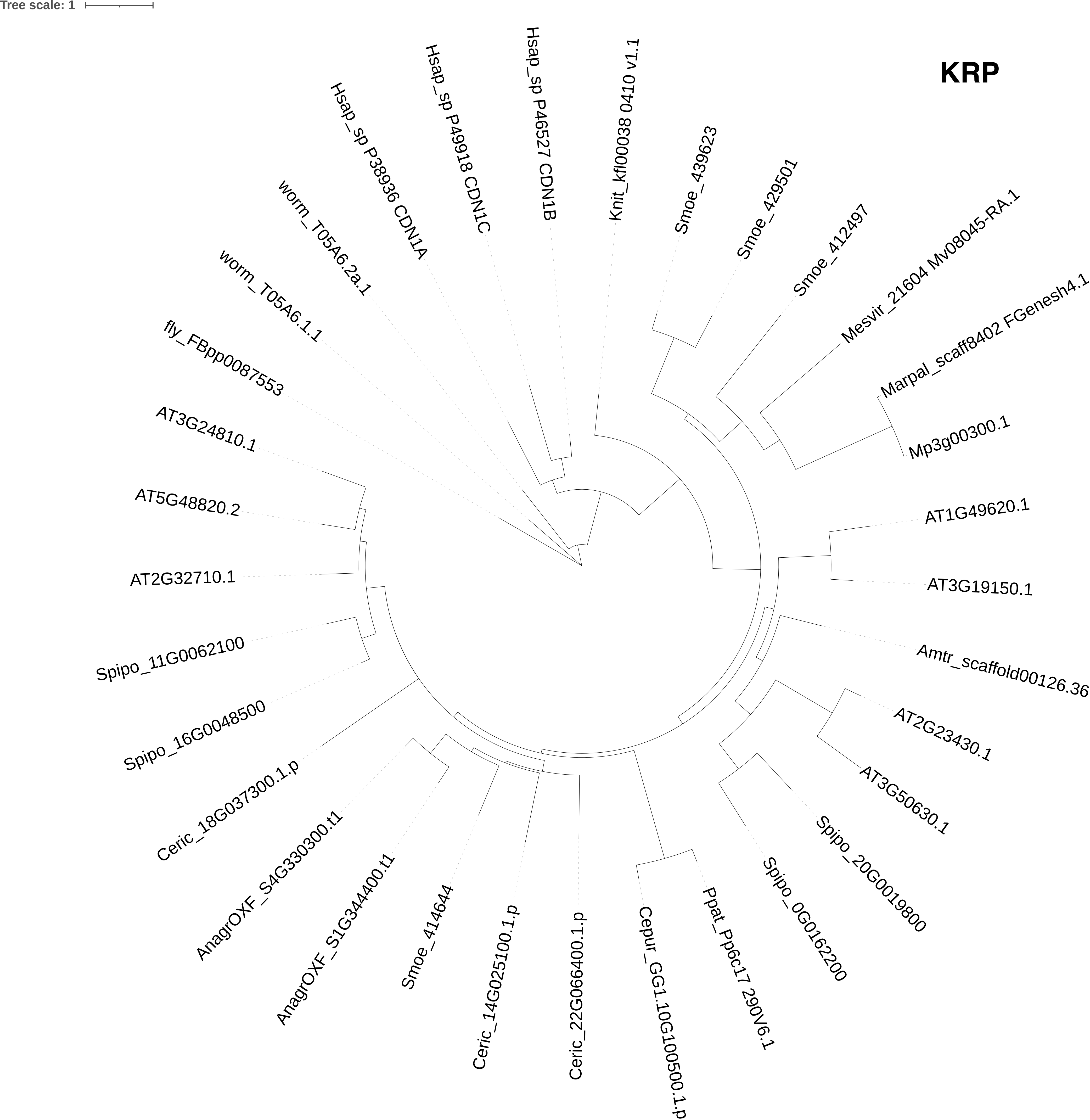

Tree scale: 1
KRP
Hsap_sp P38936 CDN1A
Hsap_sp P49918 CDN1C
Hsap_sp P46527 CDN1B
Knit_kfl00038 0410 v1.1
Smoe_439623
Smoe_429501
Smoe_412497
Mesvir_21604 Mv08045-RA.1
Marpal_scaff8402 FGenesh4.1
Mp3g00300.1
AT1G49620.1
AT3G19150.1
Amtr_scaffold00126.36
AT2G23430.1
AT3G50630.1
Spipo_20G0019800
Spipo_0G0162200
Ppat_Pp6c17 290V6.1
Cepur_GG1.10G100500.1.p
Ceric_22G066400.1.p
Ceric_14G025100.1.p
Smoe_414644
AnagrOXF_S1G344400.t1
AnagrOXF_S4G330300.t1
Ceric_18G037300.1.p
Spipo_16G0048500
Spipo_11G0062100
AT2G32710.1
AT5G48820.2
AT3G24810.1
fly_FBpp0087553
worm_T05A6.1.1
worm_T05A6.2a.1
