# Supplementary material for: A simple cell-cycle control system in Marchantia polymorpha provides a framework for understanding plant cell proliferation
Source: Plant Cell. 2026 Apr 9;38(6):koag103. doi: 10.1093/plcell/koag103 (PMC13237563; doi:10.1093/plcell/koag103)
Supplement: koag103_Supplementary_Data [file koag103_supplementary_data.zip › TPC-2025-0903R2_Suppl_Data.pdf]

Supplementary Data for Romani et al. (2026). "A simple cell-cycle control system in *Marchantia polymorpha* provides a framework for understanding plant cell proliferation".

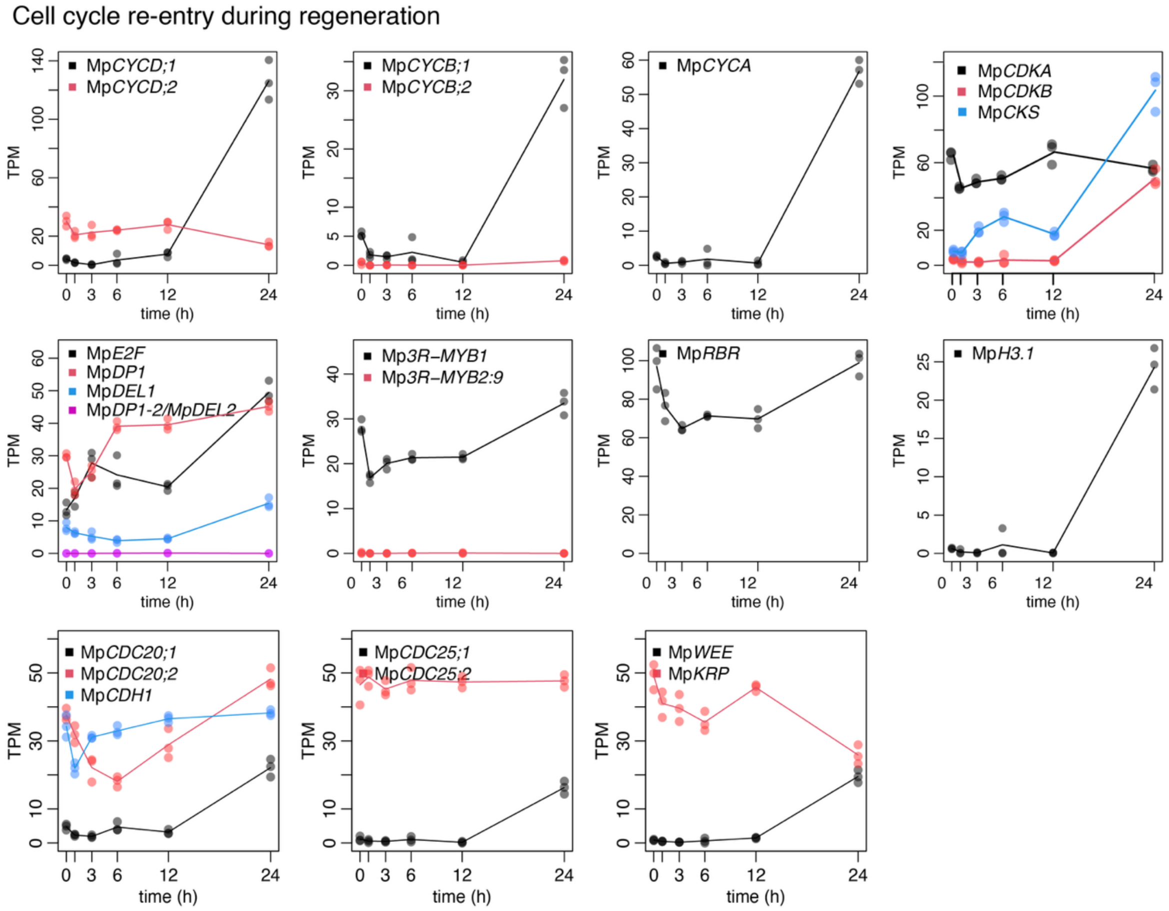

Supplementary Figure S1. Expression analysis of core cell cycle genes in Marchantia during regeneration. RNA-seq of other CDKs in regeneration (Ishida et al., 2021). Genes are grouped per families and distinguished by colour (see legend). Individual points represent biological replicates and lines averages. TPM, transcripts per million.

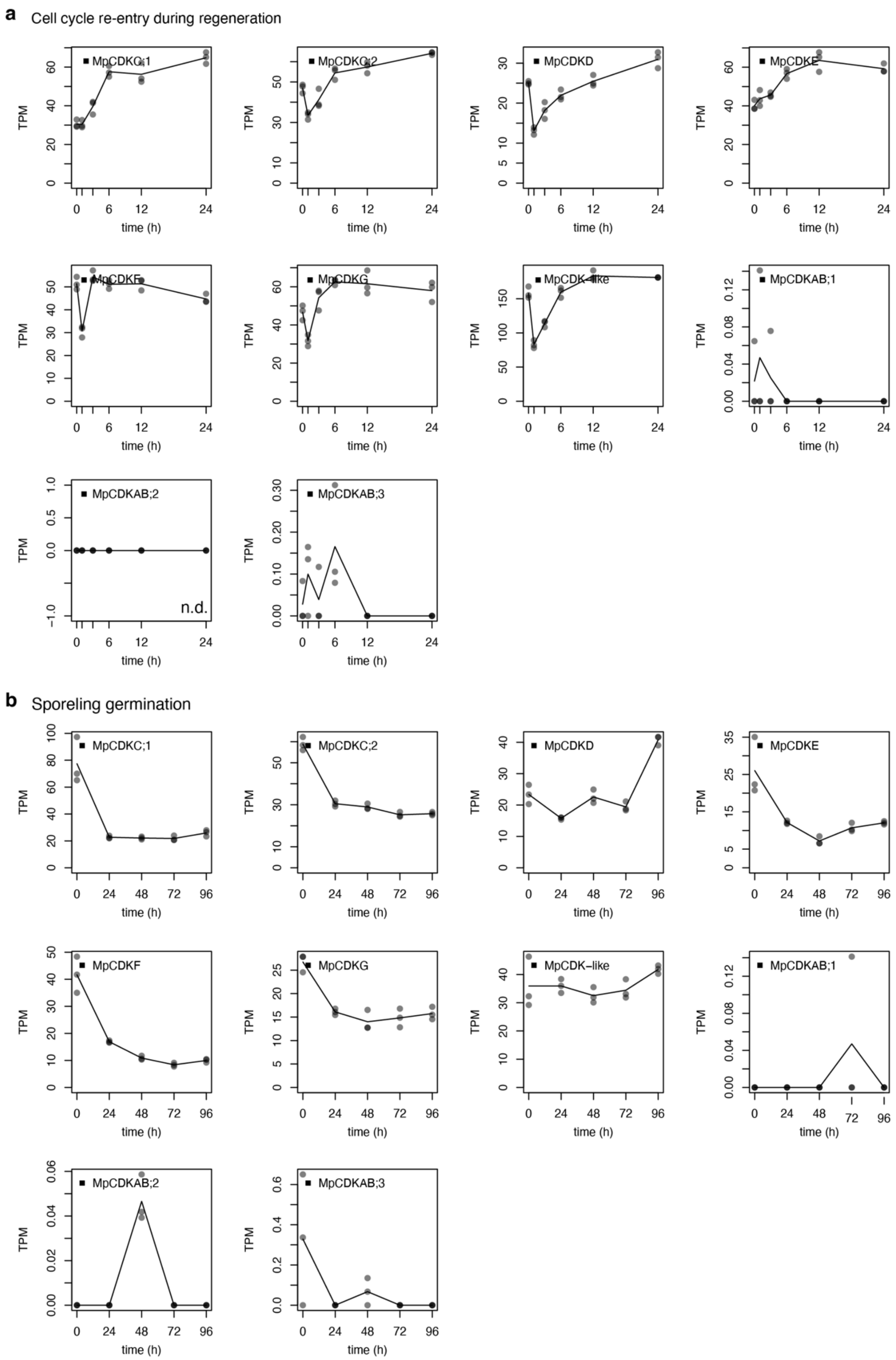

Supplementary Figure S2. Expression analysis of other CDKs in regeneration and sporeling germination in Marchantia. RNA-seq of other CDKs in regeneration and sporeling germination (Bowman et al., 2017; Ishida et al., 2021). Individual points represent biological replicates and lines averages. TPM, transcripts per million.

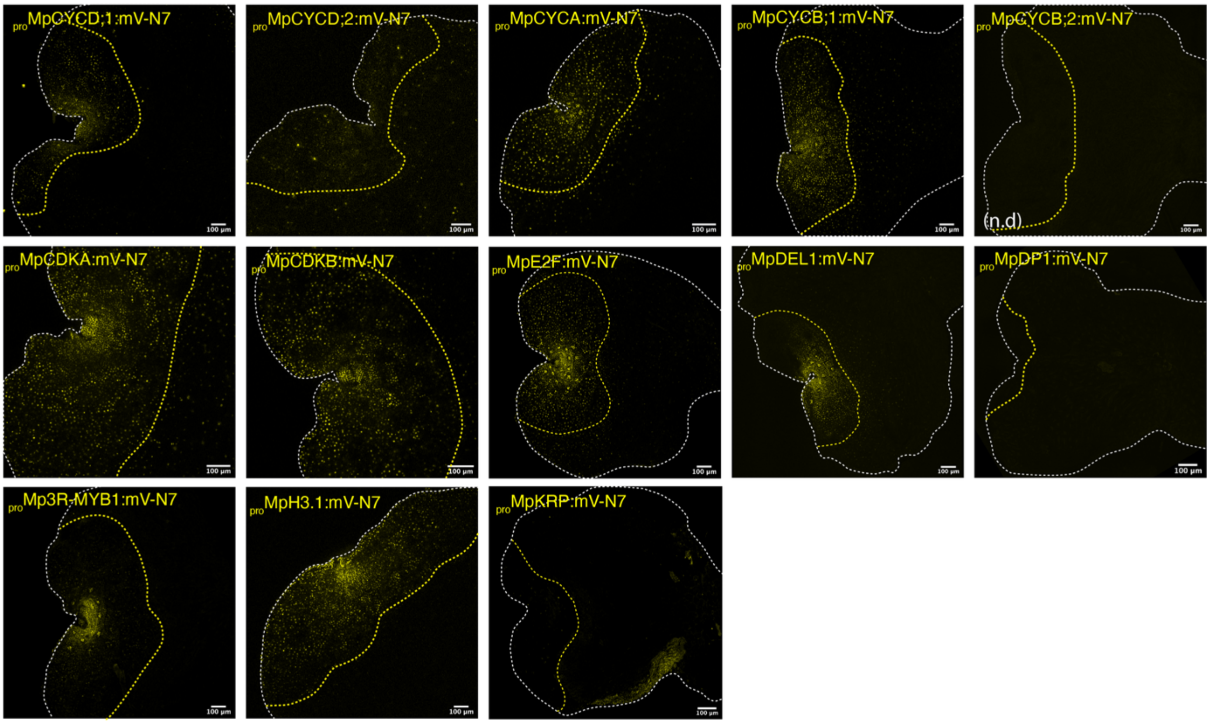

Supplementary Figure S3. Transcriptional reporters of selected cell cycle genes. Confocal images of transcriptional fluorescent reporters (mVenus, yellow) in 5-days old gemmalings expressing the indicated constructs. Images are representative of 4 independent lines obtained per construct. Reporter signal in yellow. The white dashed lines delimitate the plant outlines. The yellow dashed lines mark the boundary of the mature epidermis. Scale bar is shown in each image.

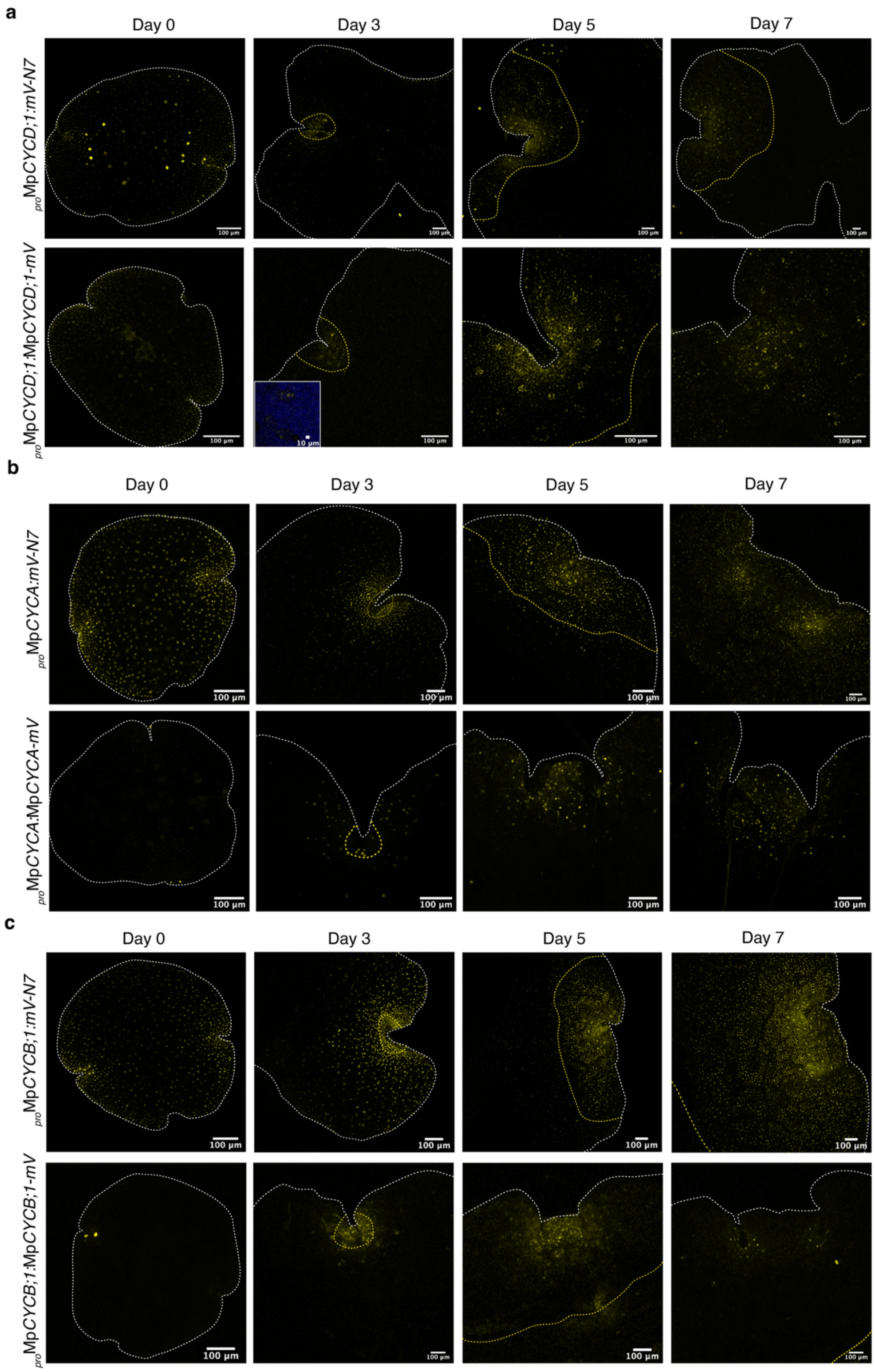

Supplementary Figure S4. Time-course of transcriptional reporters of cyclins. Time-courses of Mp*CYCD;1* (a), Mp*CYCA* (b), and Mp*CYCB;1* (c) transcriptional and translational reporters (mVenus, yellow) in Marchantia gemmallings (0, 3, 5, and 7 days) expressing the indicated constructs. Images are representative of 4 independent lines obtained per construct. The white dashed lines delimit the outlines of the plants. The yellow dashed lines mark the boundary of the mature epidermis. Scale bar is shown in each image. The square in Mp*CYCD;1* highlight a rhizoid precursor cell (chlorophyll autofluorescence in blue).

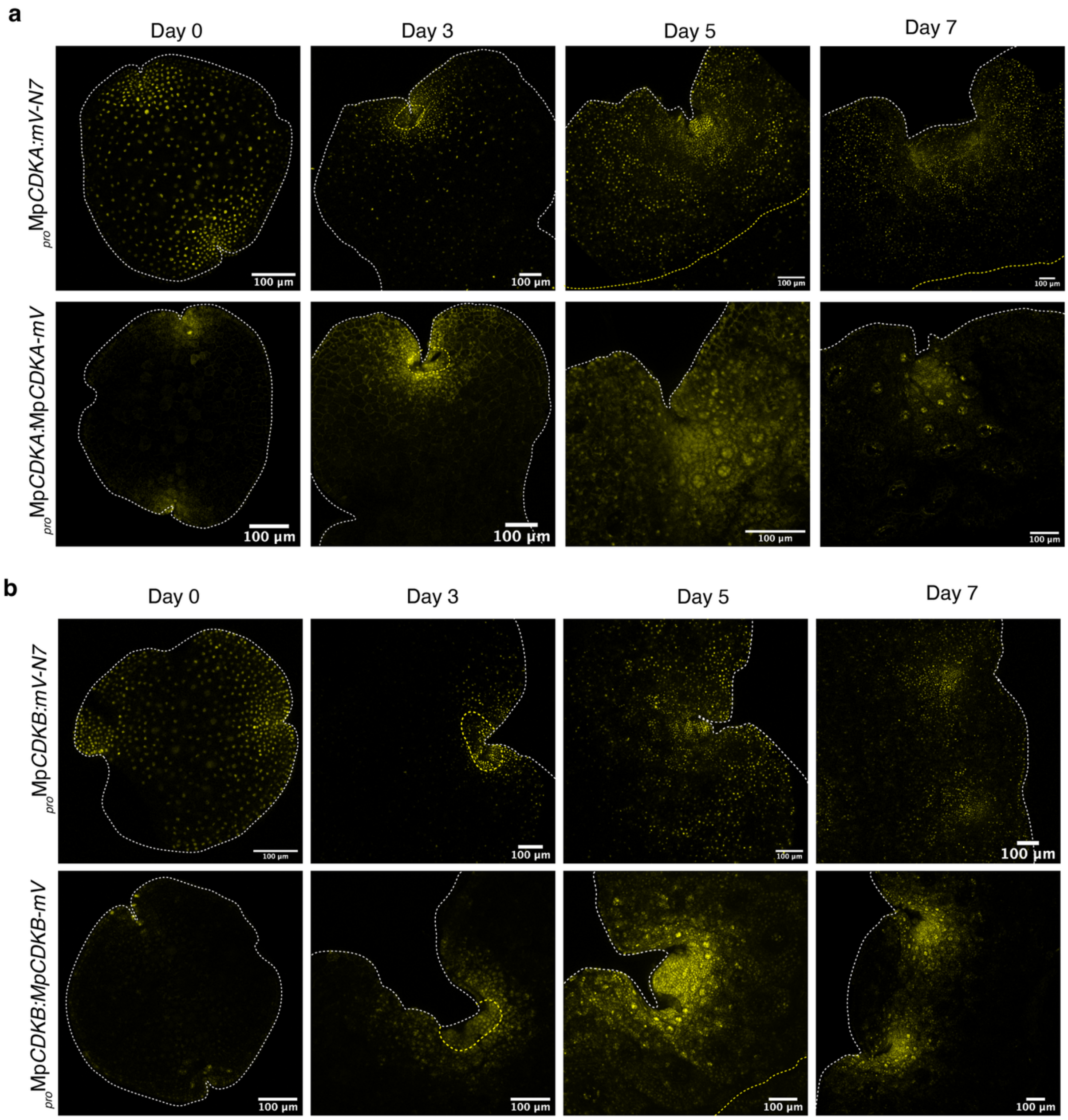

Supplementary Figure S5. Time-course of translational reporters of CDKs. Time-courses of Mp*CDKA* (a), and Mp*CDKB* (b) transcriptional and translational reporters (yellow) in Marchantia gemmallings (0, 3, 5, and 7 days) expressing the indicated constructs. Images are representative of 4 independent lines obtained per construct. The white dashed line delimitates the outline of the plant. The yellow dashed line represents the boundary between the mature epidermis and the supportive tissue of the gemmae. Scale bar is shown in each image.

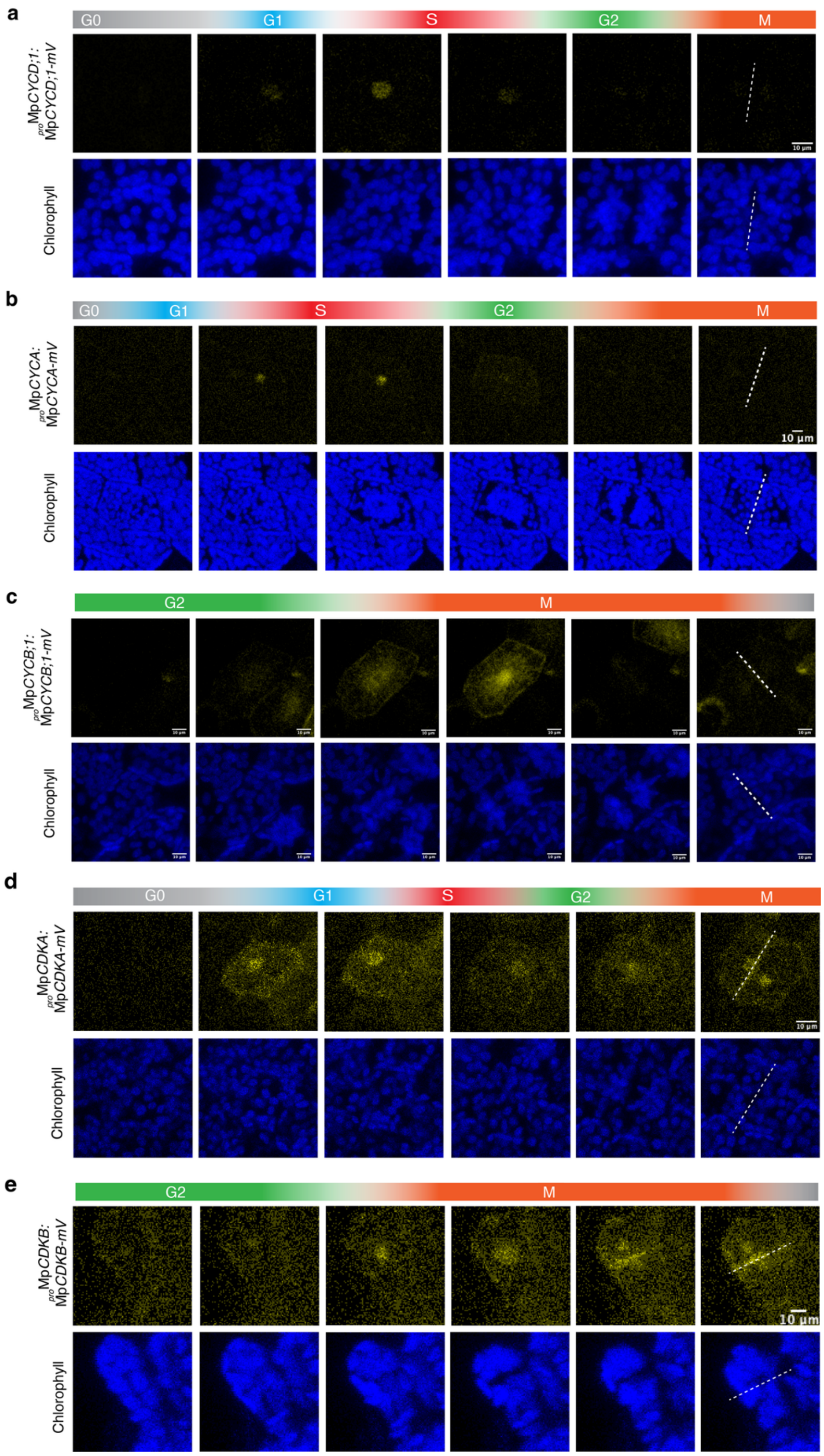

Supplementary Figure S6. Time-lapse of translational reporters of cyclins and CDKs on individual cells. a-b) Time-lapse of Mp*CYCD;1* (a) Mp*CYCA* (b), and Mp*CYCB;1* (c) Mp*CDKA* (d) and Mp*CDKB* (e) translational reporters of an individual cell dividing during regeneration (yellow). Chlorophyll (blue) is shown to visualize the cell division phases using chloroplast movement. Dashed lines indicate the new cell division plane. The full videos of the time-lapses are available at Supplementary Video 4-5. Scale bar is shown in each image.

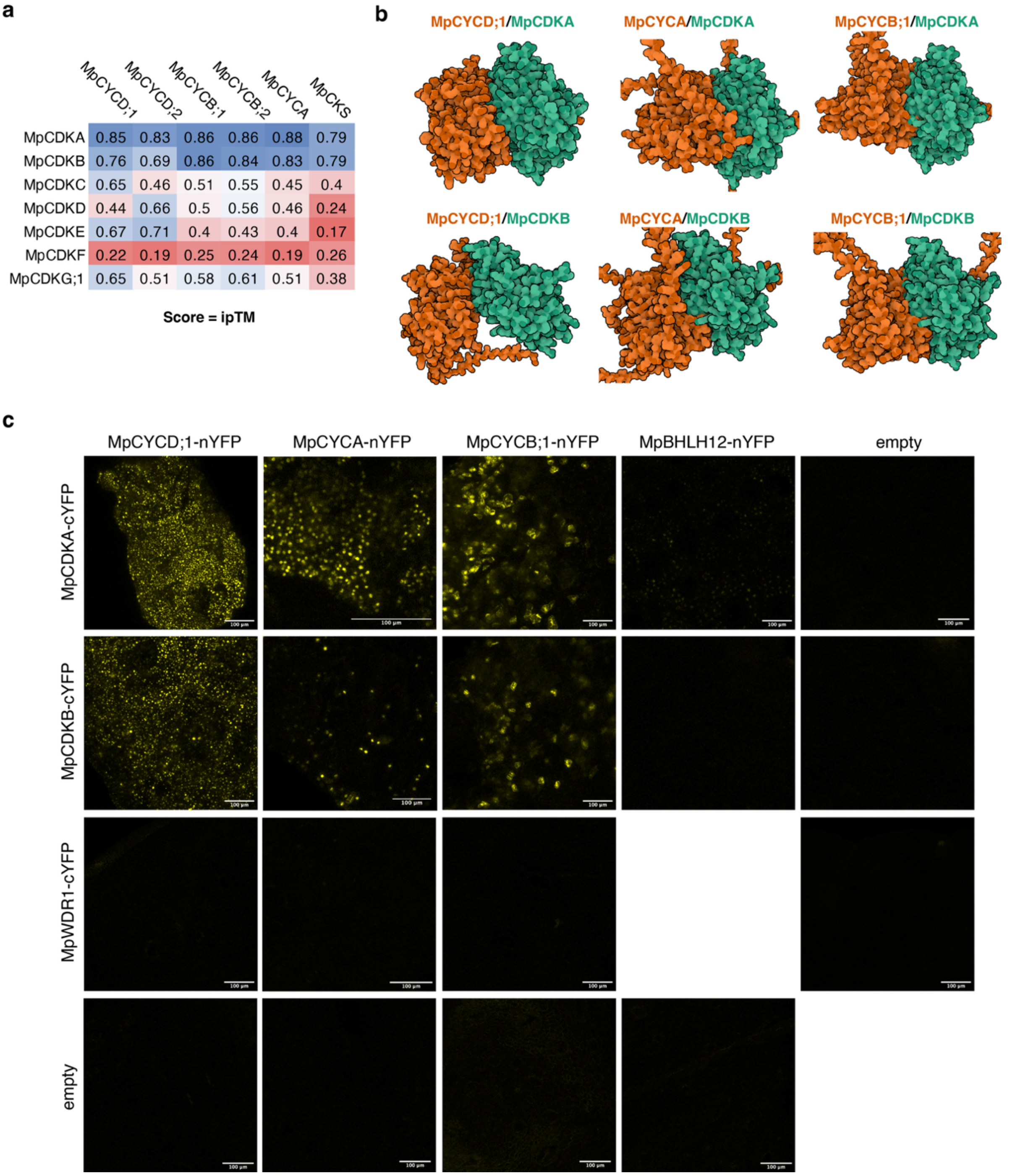

Supplementary Figure S7. Protein-protein interactions of cyclins and CDKS. (a) Interaction scores of cyclin-CDK pairs predicted using AlphaFold3. ipTM (Interface predicted template modelling). Scores > 0.8 are interpreted as a high-confidence interaction, 0.6-0.8 medium-confidence interactions, and < 0.6 as low-confidence interactions. (b) Example 3D models of the main cyclin CDK pairs. (c) Native proteins to the N-terminal fragment of YFP (columns) or the C-terminal fragment of YFP (rows) were co-transformed in Marchantia. Representative confocal images of sporelings after two weeks under selection. Reconstituted YFP fluorescence indicates direct protein-protein interactions in vivo. MpBHLH12 and MpWDR1 were used as negative controls (Kongsted et al., 2025). Scale bars = 100 µm.

a

Mp1g24670/Mp*CYCD;2*

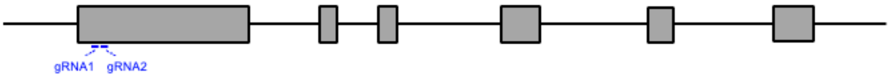

gRNA1

WT CAAGCTTCTGTCCAAGTCCAAGCCGT--------------------CGGGCGGCTCACGA
Mp*cycd;2-1*^{ge} CAAGCTTCTGTCCAAGTCCAAGCCGTCATCTCACGGCGGACTCAAGCGGGCGGCTCACGA

gRNA2

WT ACCATGCACGCCTGCCCGCTTGCGCTC-CAGAGGCCGTGGA
Mp*cycd;2-2*^{ge} ACCATGCACGCCTGCCCGCTTGCGCTCTCAGAGGCCGTGGA

b

MpCYCD;2
MCVRYFSRRRCQSSKKTFRKKLRPSPECHKLLSKSKPSGGSRDHARLPACAPEAVDLFCPESLPMLDPQSCQVSSD
AAAAAVEPSDLPTDELRLVAPMTTPSSSSSSDPGAAFNAKSPPGCNVKDGVWHDIDGMPQMLPSMGPRAADPRHGL
ADGGGWHNFCPDPGSQPGLGAVKRRGWHLETAASFISSGNSRQSNYLPCERWPVRARAPLSLPSTRTACHDRRQPT
KPSSSFRINGAMRLPDDHDFSCPSKFGEAMGILLEKETASCFQLSKPYIDGLTENSAQKLEHNELRDERRAIVNWI
FEVTRLLQLSSLSAAHAVSFLDRYLSQRFFPDAEFGANVDIRLAATACIWIAAKLEDSGAPDLSSMRELFSLDYDE
EKIRKMEVSVLKCLGWRMLAVTALDFLANMVAQLPAFGLPTHLLRPVGHRSEEIMQGLLSEVEFLAYQPSVVSMSV
MQCLLDECVPVHTASIMAAFHLALAFDVESEKVCHRTLMDLVVDPDLVCSLPSPDRVDCASSREEPSGCSQWTDAI
GHRYLLSTSTSSSLRSSEILKC*
Mp*cycd;2-1*^{ge}
MCVRYFSRRRCQSSKKTFRKKLRPSPECHKLLSKSKPSSHGGLKRAAHETMHACPLALQRPWTCFAPSRSPC*
Mp*cycd;2-2*^{ge}
MCVRYFSRRRCQSSKKTFRKKLRPSPECHKLLSKSKPSGGSRDHARLPACALRGRGLVLPRVAPHVRSTELSGLFR
CCCCSSRAV*

c

Mp3g00300/Mp*KRP*

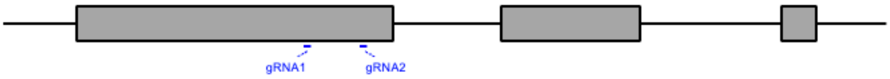

gRNA2

WT TTAGCCGACCCACGTGAGACTAAGC-G-CTCGGGGGAGCTG
Mpkrp-1^{ge} TTAGCCGACCCACGTGAGACTAAGC-GACTCGGGGGAGCTG
Mpkrp-2^{ge} TTAGCCGACCCACGTGAGACTAAGCGG-CTCGGGGGAGCTG

d

MpKRP
MGRHIQSGTRMDVAQDVRDVSLANENVVVSEQEQNFEGRSTNTQLQLQIAVSETVRSSNLVESLERKDETAKTDIE
PGCEEKQILIAEMTKPTKSEESVTARDLSTNTSDEFRTGAEPPARRRLSIGTGSTSSRVTDAEGHLNRGELVVETK
TRDEQTGASANNKRPAVSSPTITGSPVWWDANRLADPRETKRSGELMHRPRRGGIQHASTWPSFSRWESINCETRA
QQPYSVQCSLIDVRLDGISDDSTSSMQIDPVPMSRSREASPEPTVISFEESQELVHRQSLGMRTKHHRTRPASAPR
PVNDSVATCSQPDFLSWKLQIEKRNQERHGRTPPDRDFAERYNFDIHSDSPLKGRWEWEKMQQP*
Mp*krp-1*^{ge}
MGRHIQSGTRMDVAQDVRDVSLANENVVVSEQEQNFEGRSTNTQLQLQIAVSETVRSSNLVESLERKDETAKTDIE
PGCEEKQILIAEMTKPTKSEESVTARDLSTNTSDEFRTGAEPPARRRLSIGTGSTSSRVTDAEGHLNRGELVVETK
TRDEQTGASANNKRPAVSSPTITGSPVWWDANRLADPRETKRLGGADAQTSSRWHPACEYMAQFFTLGVNQLRNTG
PTALQRAVLLD*
Mp*krp-2*^{ge}
MGRHIQSGTRMDVAQDVRDVSLANENVVVSEQEQNFEGRSTNTQLQLQIAVSETVRSSNLVESLERKDETAKTDIE
PGCEEKQILIAEMTKPTKSEESVTARDLSTNTSDEFRTGAEPPARRRLSIGTGSTSSRVTDAEGHLNRGELVVETK
TRDEQTGASANNKRPAVSSPTITGSPVWWDANRLADPRETKRGSGELMHRPRRGGIQHASTWPSFSRWESINCETR
AQQPYSVQCSLI*

Supplementary Figure S8. Genotyping of Mp*cycd;2*^{ge} and Mp*krp*^{ge}. a) Structure of *Mp1g24670*/Mp*CYCD;2* locus with the position of designed guide RNA (gRNA). Exons are shown as boxes. Genotyping of genome editing alleles (bottom). gRNA sequence is in blue. Deleted or inserted bases are indicated in red. b) Wild-type (Mp*CYCD;2*) and mutant protein sequences deduced from the genomic DNA sequences are indicated. c-d) Same for *Mp3g00300*/Mp*KRP*. Sequences different from WT are in red. Asterisks indicate translational termination.

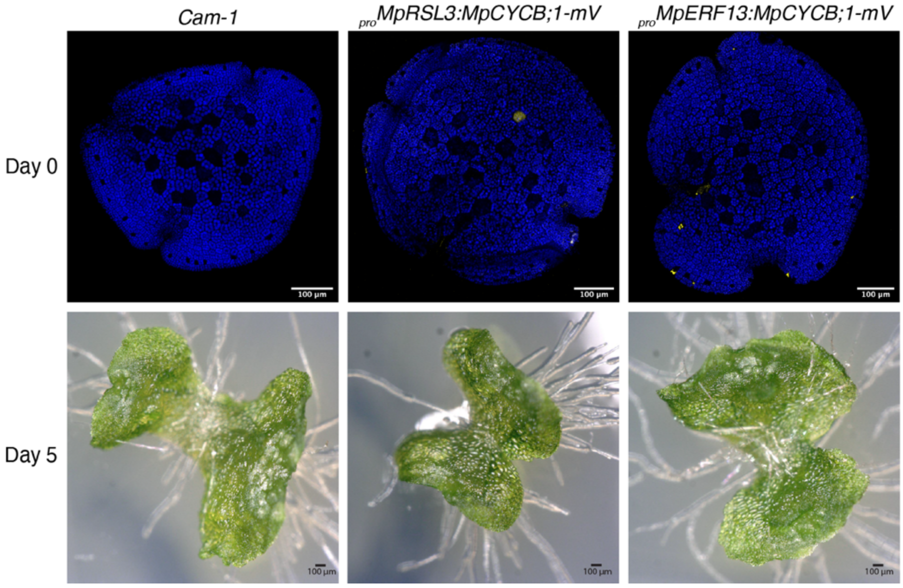

Supplementary Figure S9. Cell-type specific overexpression of Mp*CYCB;1* using cell-types specific promoters. Gemmalings expressing $_{pro}$Mp*RSL3*:Mp*CYCB;1*-mVenus (rhizoid precursors specific) and $_{pro}$Mp*ERF13*:Mp*CYCB;1-mVenus* (oil bodies specific). Wild-type *Cam-1* is used as a control. Confocal full-stack images of representative individual gemma are shown with mVenus (yellow) and chlorophyll (blue) channels merged for 0 days gemma (top). The mVenus channel is not shown in the wild-type. Representative picture of 5-days old gemmalings (bottom). Scale bar lengths are indicated in the pictures.

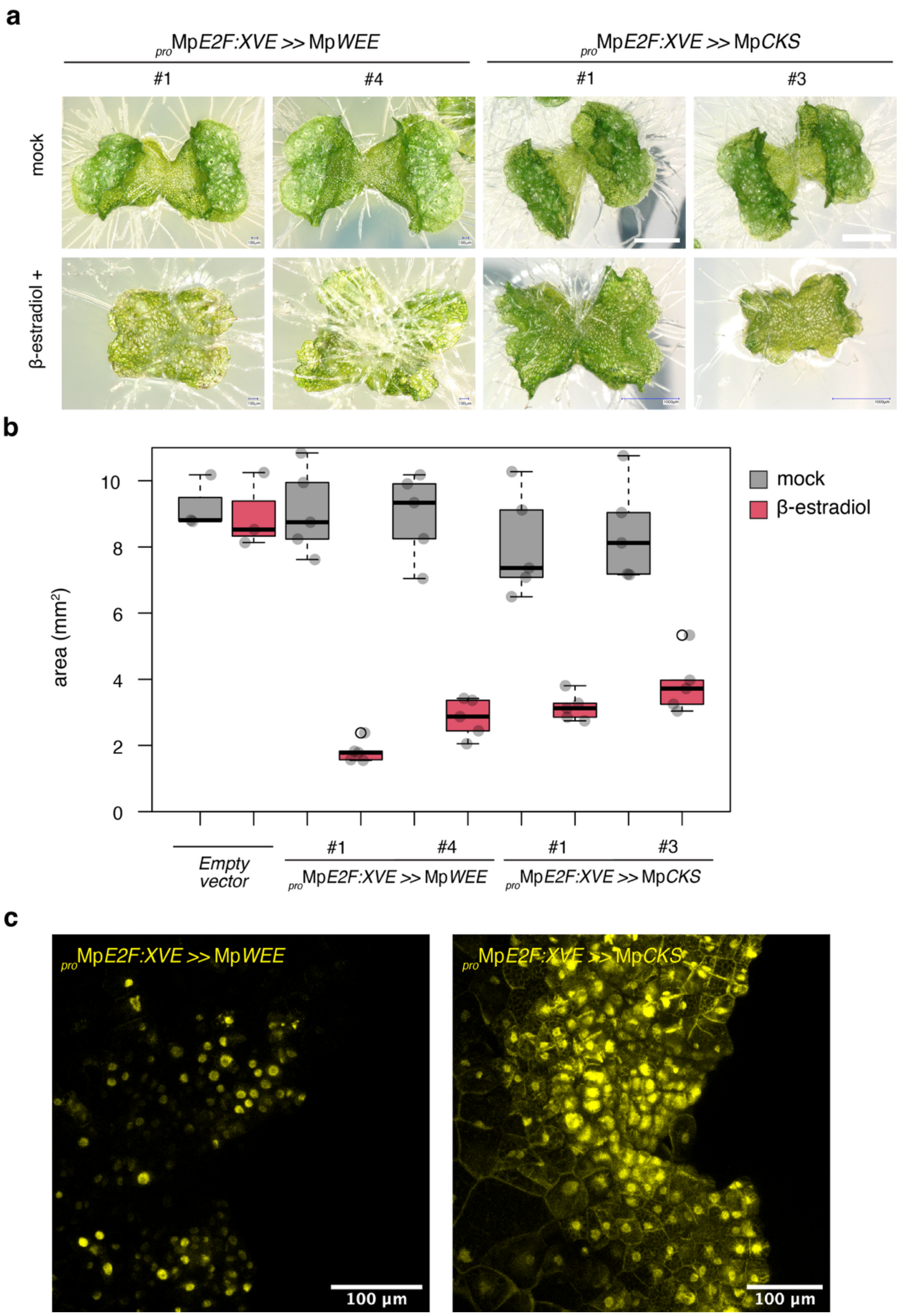

Supplementary Figure S10. Inducible overexpression of MpWEE and MpCKS. a) Representative pictures of 7-days-old gemmalings of transgenic plants with inducible overexpression of plants expressing *pro*Mp*E2F:XVE* >> Mp*WEE* or Mp*CKS* grown with or without induction. b) Boxplot of plant area of plants of the same age are shown, including a control using transgenic plants transformed with the empty backbone (n=5). c) Representative confocal imaging of 4-days-old gemmaling of *pro*Mp*E2F:XVE* >> Mp*WEE* or Mp*CKS* in inductive conditions.

SUPPLEMENTAL VIDEOS

Supplementary Video S1. *pro*Mp*CYCD;1*:Mp*CYCD;1-mVenus* time-lapse during regeneration. mVenus in yellow and chlorophyll autofluorescence in blue. Scale bar is indicated in the video.

Supplementary Video S2. *pro*Mp*CYCA*:Mp*CYCA-mVen*us time-lapse during regeneration. mVenus in yellow and chlorophyll autofluorescence in blue. Scale bar is indicated in the video.

Supplementary Video S3. *pro*Mp*CYCB;1*:Mp*CYCB;1-mVenus* time-lapse during regeneration. mVenus in yellow and chlorophyll autofluorescence in blue. Scale bar is indicated in the video.

Supplementary Video S4. *pro*Mp*CDKA*:Mp*CDKA-mVenus* time-lapse during regeneration. mVenus in yellow and chlorophyll autofluorescence in blue. Scale bar is indicated in the video.

Supplementary Video S5. *pro*Mp*CDKB*:Mp*CDKB-mVenus* time-lapse during regeneration. mVenus in yellow and chlorophyll autofluorescence in blue. Scale bar is indicated in the video.

Supplementary Video S6. *pro35S*:Mp*CYCB1-mVenus* time-lapse during gemmallings development. mVenus in yellow. Scale bar is indicated in the video.

Supplementary Video S7. *pro*Mp*CYCB;1*:Mp*CYCB1-mVenus* time-lapse during gemmallings development. mVenus in yellow. Scale bar is indicated in the video.

SUPPLEMENTAL TABLES

Supplemental Table S1. Complete list of scRNA-seq cluster-specific markers and gene annotations.

Supplemental Table S2. Vectors, cloning, primers, and details of gene IDs used.

Supplemental Dataset S1. Supporting information for phylogenetic analysis of cell-cycle proteins. (a) Phylogeny of Cyclins. (b) Phylogeny of the CDK and PSTAIRE motifs. (c) Phylogeny of CKS proteins. (d) Phylogeny of E2F/DP/DEL proteins. (e) Phylogeny of RBR. (f) Phylogeny of 3R-MYBs and analysis of sequence repeats. (g) Phylogeny of CDC20/CDH1 proteins. (h) Phylogeny of CDC25 proteins. (i) Phylogeny of WEE proteins. (j) Phylogeny of KRP proteins. FAST files corresponding to protein alignment and newick tree files.

Supplemental Dataset S2. Gene bank file for *pBy13,* Golden-gate compatible binary plasmid acceptor for XVE inducible overexpression with Hygromycin resistance
cassette.

Supplemental Dataset S3. Gene bank file for *pBy23,* Golden-gate compatible binary plasmid acceptor for XVE inducible overexpression with Chlorsulfuron resistance
cassette.

REFERENCES

Ishida, S., Suzuki, H., Iwaki, A., Kawamura, S., Yamaoka, S., Kojima, M., Takebayashi, Y., Yamaguchi, K., Shigenobu, S., Sakakibara, H., Kohchi, T., and Nishihama, R. (2022). Diminished Auxin Signaling Triggers Cellular Reprogramming by Inducing a Regeneration Factor in the Liverwort Marchantia polymorpha. Plant Cell Physiol **63,** 384-400.

Bowman, J.L., Kohchi, T., Yamato, K.T., Jenkins, J., Shu, S., Ishizaki, K., Yamaoka, S., Nishihama, R., Nakamura, Y., Berger, F., Adam, C., Aki, S.S., Althoff, F., Araki, T., Arteaga-Vazquez, M.A., Balasubrmanian, S., Barry, K., Bauer, D., Boehm, C.R., Briginshaw, L., Caballero-Perez, J., Catarino, B., Chen, F., Chiyoda, S., Chovatia, M., Davies, K.M., Delmans, M., Demura, T., Dierschke, T., Dolan, L., Dorantes-Acosta, A.E., Eklund, D.M., Florent, S.N., Flores-Sandoval, E., Fujiyama, A., Fukuzawa, H., Galik, B., Grimanelli, D., Grimwood, J., Grossniklaus, U., Hamada, T., Haseloff, J., Hetherington, A.J., Higo, A., Hirakawa, Y., Hundley, H.N., Ikeda, Y., Inoue, K., Inoue, S.I., Ishida, S., Jia, Q., Kakita, M., Kanazawa, T., Kawai, Y., Kawashima, T., Kennedy, M., Kinose, K., Kinoshita, T., Kohara, Y., Koide, E., Komatsu, K., Kopischke, S., Kubo, M., Kyozuka, J., Lagercrantz, U., Lin, S.S., Lindquist, E., Lipzen, A.M., Lu, C.W., De Luna, E., Martienssen, R.A., Minamino, N., Mizutani, M., Mizutani, M., Mochizuki, N., Monte, I., Mosher, R., Nagasaki, H., Nakagami, H., Naramoto, S., Nishitani, K., Ohtani, M., Okamoto, T., Okumura, M., Phillips, J., Pollak, B., Reinders, A., Rovekamp, M., Sano, R., Sawa, S., Schmid, M.W., Shirakawa, M., Solano, R., Spunde, A., Suetsugu, N., Sugano, S., Sugiyama, A., Sun, R., Suzuki, Y., Takenaka, M., Takezawa, D., Tomogane, H., Tsuzuki, M., Ueda, T., Umeda, M., Ward, J.M., Watanabe, Y., Yazaki, K., Yokoyama, R., Yoshitake, Y., Yotsui, I., Zachgo, S., and Schmutz, J. (2017). Insights into Land Plant Evolution Garnered from the Marchantia polymorpha Genome. Cell **171,** 287-304 e215.

Kongsted, T.E., Romani, F., Airoldi, C.A., Haseloff, J., Glover, B.J. (2025) Replicated repurposing of an ancestral transcriptional complex in land plants. BioRxiv, doi: 10.1101/2025.04.25.650595.
