# Supplementary material for: A simple cell-cycle control system in Marchantia polymorpha provides a framework for understanding plant cell proliferation
Source: Plant Cell. 2026 Apr 9;38(6):koag103. doi: 10.1093/plcell/koag103 (PMC13237563; doi:10.1093/plcell/koag103)
Supplement: koag103_Supplementary_Data [file koag103_supplementary_data.zip › TPC-2025-0903R2_Supplementary Video Legends.pdf]

Supplementary Video Legends

Suppl. Video 1. *pro*Mp*CYCD;1*:Mp*CYCD;1-mVenus* time-lapse during regeneration. mVenus in yellow and chlorophyll autofluorescence in blue. Scale bar is indicated in the video.

Suppl. Video 2. *pro*Mp*CYCA*:Mp*CYCA-mVen*us time-lapse during regeneration. mVenus in yellow and chlorophyll autofluorescence in blue. Scale bar is indicated in the video.

Suppl. Video 3. *pro*Mp*CYCB;1*:Mp*CYCB;1-mVenus* time-lapse during regeneration. mVenus in yellow and chlorophyll autofluorescence in blue. Scale bar is indicated in the video.

Suppl. Video 4. *pro*Mp*CDKA*:Mp*CDKA-mVenus* time-lapse during regeneration. mVenus in yellow and chlorophyll autofluorescence in blue. Scale bar is indicated in the video.

Suppl. Video 5. *pro*Mp*CDKB*:Mp*CDKB-mVenus* time-lapse during regeneration. mVenus in yellow and chlorophyll autofluorescence in blue. Scale bar is indicated in the video.

Suppl. Video 6. *pro35S*:Mp*CYCB1-mVenus* time-lapse during gemmallings development. mVenus in yellow. Scale bar is indicated in the video.

Suppl. Video 7. *pro*Mp*CYCB;1*:Mp*CYCB1-mVenus* time-lapse during gemmallings development. mVenus in yellow. Scale bar is indicated in the video.
